# Availability and representativeness of freshwater macroinvertebrate data to enable Europe-wide assessment of biodiversity trends

**DOI:** 10.1371/journal.pone.0354723

**Published:** 2026-08-13

**Authors:** Iris R. Pit, Anna Sobek, Annemarie P. van Wezel, Sofia A. Wikström, W. Daniel Kissling, Florian Altermatt, Libuše Barešová, José Barquín, Angela Boggero, Miguel Cañedo-Argüelles, Zoltán Csabai, Elvira de Eyto, Alain Dohet, Stina Drakare, Michael J. Dunbar, Judy England, Tor E. Eriksen, Vesela Evtimova, Mathieu Floury, Marie Anne Eurie Forio, Riccardo Fornaroli, Peter Haase, Kaisa-Leena Huttunen, Richard K. Johnson, Ioannis Karaouzas, Lenka Kuglerová, Alex Laini, Aitor Larrañaga, Patrick Leitner, Armin W. Lorenz, Dāvis Ozoliņš, Petr Pařil, Francesco Polazzo, Jes Jessen Rasmussen, Andreu Rico, Jouko Rissanen, Ralf B. Schäfer, Astrid Schmidt-Kloiber, Alberto Scotti, James S. Sinclair, Agnija Skuja, Henn Timm, Iakovos Tziortzis, Frank Van de Meutter, Rudy Vannevel, Gábor Várbíró, Gaute Velle, Piet F. M. Verdonschot

**Affiliations:** 1 Institute for Biodiversity and Ecosystem Dynamics, University of Amsterdam, Amsterdam, The Netherlands; 2 Department of Environmental Science, Stockholm University, Stockholm, Sweden; 3 Deltares, Boussinesqweg 1, Delft, The Netherlands; 4 Faculty of Geosciences, Utrecht University, Princetonlaan 8a, Utrecht, The Netherlands; 5 Baltic Sea Centre, Stockholm University, Stockholm, Sweden; 6 Department of Evolutionary Biology and Environmental Studies, Faculty of Science, University of Zurich, Winterthurerstrasse 190, Zurich, Switzerland; 7 Department of Aquatic Ecology, Eawag: Swiss Federal Institute of Aquatic Science and Technology, Überlandstrasse 133, Dübendorf, Switzerland; 8 Czech Hydrometeorological Institute, Na Šabatce 17, Praha, Czech Republic; 9 Environmental Hydraulics Institute (IHCantabria), Universidad de Cantabria, Parque Científico y Tecnológico de Cantabria, C/Isabel Torres nº 15, Santander, Cantabria, Spain; 10 National Research Council - Water Research Institute (CNR-IRSA), C.so Tonolli 50, Verbania Pallanza, Italy; 11 FEHM-Lab (Freshwater Ecology, Hydrology and Management), SHE-2, Institute of Environmental Assessment and Water Research (IDAEA), CSIC, Carrer de Jordi Girona, 18-26, Barcelona, Spain; 12 Department of Hydrobiology, University of Pécs, Ifjúság útja 6, Pécs, Hungary; 13 HUN-REN Balaton Limnological Research Institute, Klebelsberg Kuno 3, Tihany, Hungary; 14 Marine Institute, Furnace, Newport, Co. Mayo, Ireland; 15 Luxembourg Institute of Science and Technology (LIST), 5 Av. des Hauts-Fourneaux, Esch-Belval Esch-sur-Alzette, Luxembourg; 16 Swedish University of Agricultural Sciences (SLU), Department of Aquatic Sciences and Assessment, Uppsala, Sweden; 17 Environment Agency, Horizon House, Deanery Rd, Bristol, United Kingdom; 18 Norwegian Institute for Water Research (NIVA), Økernveien 94, Oslo, Norway; 19 Institute of Biodiversity and Ecosystem Research, Bulgarian Academy of Sciences, 1 Tsar Osvoboditel Blvd., Sofia, Bulgaria; 20 INRAE, UR RiverLy, Centre de Lyon-Villeurbanne, Villeurbanne, France; 21 Department of Animal Sciences and Aquatic Ecology, Ghent University, Coupure Links 653, Ghent, Belgium; 22 Dipartimento di Scienze dell’Ambiente e della Terra, Università di Milano-Bicocca, Piazza della scienza 1, Milano, Italy; 23 Department of River Ecology and Conservation, Senckenberg Research Institute and Natural History Museum Frankfurt, Gelnhausen, Germany; 24 Department of Aquatic Ecology, Faculty for Biology, University of Duisburg-Essen, Universitätsstr. 5, Essen, Germany; 25 Ecology and Genetics Research Unit, University of Oulu, Oulu, Finland; 26 Finnish Environment Institute, Oulu, Finland; 27 Institute of Marine Biological Resources and Inland Waters, Hellenic Centre for Marine Research, 46.7km Athens-Sounio Av., Anavyssos, Greece; 28 Swedish University of Agricultural Sciences, Dept. of Forest Ecology and Management, Skogsmarksgränd 17, Umeå, Sweden; 29 Dipartimento di Scienze della Vita e Biologia dei Sistemi, Università di Torino, via Accademia Albertina 13, Torino, Italy; 30 Department of Plant Biology and Ecology, University of the Basque Country, Leioa, Spain; 31 University of Natural Resources and Life Sciences (BOKU Vienna), Institute of Hydrobiology and Aquatic Ecosystem Management, Vienna, Austria; 32 Institute of Biology, Faculty of Medicine and Life Sciences, University of Latvia, Riga, Latvia; 33 Department of Botany and Zoology, Faculty of Science, Masaryk University, Brno, Czech Republic; 34 Aarhus University, Department of Ecoscience, Aarhus, Denmark; 35 Cavanilles Institute of Biodiversity and Evolutionary Biology, University of Valencia, c/ Catedrático José Beltrán 2, Paterna, Valencia, Spain; 36 Finnish Environment Institute (Syke), Latokartanonkaari 11, Helsinki, Finland; 37 Research Center One Health Ruhr of the University Alliance Ruhr, Faculty of Biology, University of Duisburg-Essen, Universitätsstrasse 2, Essen, Germany; 38 Institute for Alpine Environment, EURAC Research - Drususallee 1, Bozen, Italy; 39 APEM Ltd, Riverview, A17 Embankment Business Park, Heaton Mersey, Stockport, United Kingdom; 40 Chair of Hydrobiology and Fishery, Centre for Limnology, Estonian University of Life Sciences, Elva vald, Estonia; 41 Water Development Department, Ministry of Agriculture, Rural Development and Environment, Nicosia, Cyprus; 42 Research Institute for Nature and Forest (INBO), Havenlaan 88 bus 73, Brussel, Belgium; 43 Flanders Environment Agency, Dr. de Moorstraat 24-26, B-9300 Aalst, Belgium; 44 HUN-REN Centre for Ecological Research, Institute of Aquatic Ecology, Bem tér 18/c, Debrecen, Hungary; 45 NORCE Norwegian Research Centre, Laboratory for Freshwater Ecology and Inland Fisheries, Bergen, Norway; 46 Wageningen Environmental Research, Wageningen, University and Research, Wageningen, The Netherlands; University of Ferrara: Universita degli Studi di Ferrara, ITALY

## Abstract

Freshwater ecosystems, though covering less than 1% of Earth’s surface, harbor around 10% of all species. Pressures like pollution, water extraction, and habitat loss can exhibit complex interactions in their effects on freshwater biodiversity. Understanding the effects of these pressures on ecosystem services and functions of freshwater systems is crucial for effective environmental policies, and asks for availability of species data. We therefore compiled European monitoring data on macroinvertebrates from European, national, and regional biomonitoring and analysed their representativeness with respect to river size, land use as well as their suitability to derive biodiversity trends. The dataset consists of over 2.3 million macroinvertebrate abundance records from 2010 to 2015, spanning 28 European countries. Geographically, data are concentrated in central Europe and England and are not evenly representative across space or river types. They vary regarding reporting units, general sampling methods, and taxonomic levels (mainly species level). These differences hamper European-scale biodiversity comparisons. The WFD primarily focuses on assessing the “ecological status” of water bodies, which can be achieved using a menu of methods and many biodiversity research questions require improvements in harmonization of taxonomic resolutions and in method standardization to enable accurate transnational comparisons. This study emphasizes the need for improved European coordination and support in collecting, validating, exchanging, and sharing freshwater biodiversity data to enable meaningful comparisons among countries. It also delivers an open-access database that enables analyses of trends and ecological scenario development, offering further opportunities to enhance freshwater biodiversity conservation and management.

## Introduction

Freshwater ecosystems cover less than 1% of the Earth’s surface but host around 10% of global species [1]. They provide essential ecosystem services, such as drinking water, food, energy, industrial support, and recreation [2]. However, they are affected by multiple pressures, caused, e.g., by urban and agricultural land use, water extraction and hydropower generation [3,4]. Consequently, the biodiversity of freshwater ecosystems is declining faster than that of any other ecosystem [5–8]. Understanding how such pressures impact ecosystem services and ecological functions is essential for implementing effective environmental policies [3], and requires reliable and comprehensive data to enable a clear understanding of the underlying causes of biodiversity decline [9].

International efforts to curb biodiversity loss include the Intergovernmental Platform on Biodiversity and Ecosystem Services (IPBES), conservation strategies such as the Convention on Biological Diversity (CBD), the UN Sustainable Development Goals (SDGs) and the Kunming-Montreal Global Biodiversity Framework (GBF; [10]). In Europe the EU Water Framework Directive (WFD), the designation of Natura 2000 sites in accordance with the EU Birds and Habitats directives [11,12] and the recently approved EU Nature Restoration Law are of relevance. However, robust evidence and large-scale field studies examining the impact of multiple stressors on freshwater biodiversity remain relatively limited, but are needed to develop generalizable methodologies for its protection [7]. Achieving freshwater targets still poses challenges across river basins in Europe [13–15].

For successful conservation, policies and management information on the biodiversity status requiring biological monitoring, are critical [16]. Macroinvertebrates, which are abundant in freshwater systems, are frequently used as environmental, ecological and biodiversity indicators, because they are easy to collect and identify, and are largely confined to a river stretch [17,18]. Many biodiversity monitoring programs suffer from incomplete taxonomic and geographical coverage [16]. Additionally, differences in sampling methods, limited data integration across scales, and insufficient resources result in incompatible, biased and fragmented datasets [19]. To effectively prioritize mitigation measures, a better insight into the associations between species abundance with various stressors and their combinations will be mandatory [20]. When the focus is on a continental scale like Europe, this will create notable variation in the data underlying the assessments. Although ecological quality ratios (EQRs) suffice to assess ecological status as defined by the EQR methodology relative to a reference condition, they are not flexible and adjustable to assess specific stressors [21]. This limitation becomes particularly apparent in cross-boundary analyses, because different countries and regions calculate EQR relative to their own reference conditions [22]. Thus, on a national level, the EQR methodology as such works, but for transboundary analyses combined with attribution to stressors, underlying species-specific abundance data are needed.

Macroinvertebrate field data are produced by many individual scientists and institutions across Europe. These data are currently only available to a limited extent and scattered across multiple sources and databases. This may be due to (1) the costs and benefits of sharing data [23], (2) different forms of accessibility of data (e.g., supplementary files, open data repository, papers, integrated narrative, and data publishing) [24], (3) lack of metadata and quality control, and (4) other issues such as uncertainty about copyright and licensing and perceived loss of control [25]. With the widespread acceptance of the FAIR (Findable, Accessible, Interoperable, Reusable) data principles, the requirements and standards relating to published data have changed. Researchers are encouraged to ensure that upon completion of a research project, raw data, enriched metadata, processed data, software, codes, and associated material are stored in accordance with the FAIR data principles. This is to safeguard that data are valued as publishable and citable products of research [26].

Here, we aimed to estimate the availability and assess the representativeness of macroinvertebrate data in European freshwaters, and to evaluate its potential for enabling transboundary biodiversity trend analyses. For this, we compiled macroinvertebrate abundance data for the period 2010–2015 from databases, national and European authorities, and by contacting scientists. Data contributors answered a questionnaire with regards to sampling methods to provide a transparent and coherent overview of the monitoring data. Our findings show the (dis)similarities in data availability and coverage among EU member states, and we identify what is needed to accumulate sufficient and reliable data to effectively evaluate biodiversity trends at a regional scale across Europe.

## Materials and methods

### General approach

The initial phase of dataset construction involved acquiring biological raw data relevant to the Water Framework Directive (WFD) through the WISE-WFD database and EEA. While WISE provides raw data for water quality parameters, the biological data flow (WISE-2) only contains EQR values and not raw species abundance data [22], and contact information was largely restricted. Subsequent searches were conducted on global (biodiversity) platforms like GBIF and DataONE, followed by a thorough examination of 39 (inter)national databases including ICPDR (danubesurvey.org), ICPER (ikse-mkol.org), Naiades (naiades.eaufrance.fr), GFBio (www.gfbio.org) across Europe (S1 Table), and including journal repositories (e.g., [27]) to identify pertinent datasets. When data access was restricted or methods were unclear, we contacted the data owner.

As a final step, outreach efforts were made to engage with governmental institutions and scientists across Europe, seeking their further assistance in providing the data directly or facilitating connections with potential data-holders. A total of 50 scientists and governmental institutions were selected and contacted, based on their relevance to the data search and to ensure broad geographic coverage across Europe. This selection was made to maximize the potential for data retrieval, follow-up inquiries, or further communication, although not all requests received a response.

We then examined the details of the metadata (Table 1) to evaluate the level of detail in the raw macroinvertebrate abundance records. The compiled macroinvertebrate dataset focuses primarily on rivers, including some representation of lakes. It provides pan-European coverage, including EU member states, Switzerland, Serbia, Norway, and the UK. For consistency and to ensure inclusion of a sufficient volume of data, we restricted the dataset to the period 2010–2015. This timeframe also aligns with the first WFD cycle, providing a robust foundation for further assessments of impact of stressors. All available data within the 2010–2015 timeframe were included, ranging from single observations at individual sites to multi-year time series, with no restrictions given on the amount. Consequently, data coverage and temporal resolution vary considerably among countries, reflecting differences in data availability rather than deliberate selection criteria.

**Table 1:** Details of metadata from individual data sets.

*Category*	*Details*
*Location*	*Information on the geographical location (e.g., coordinates)*
*Methods*	*Date, equipment used, the number of subsamples, followed protocol or scientific guidelines, included in the WFD monitoring*
*Results*	*Individual counts per metric or sample, identified taxa*
*Quality*	*Use of established protocols or scientific guidelines*

### Data alignment

Taxon names and taxonomic level were harmonized using the AQEM/STAR taxa list from freshwaterecology.info [28]. Abundance records with taxonomic levels consisting of species, genus, tribe, subfamily, family, order, or class were included in the dataset. We discarded abundance records where (1) taxon names were not listed on freshwaterecology.info in order to exclude terrestrial and marine taxa, (2) individual count values were 0 or absent, (3) records were duplicates based on location, time, taxon name and individual count value, or (4) this was suggested by the data contributor, because of errors or outdated sampling methods.

### Geographical coverage characteristics

To assess the land-use context and river size of the abundance records, the data was processed using Quantum GIS [29]. As a proxy for river size, the Strahler stream order was assigned to each record [30] based on the EEA Catchments and Rivers Network System (ECRINS) v1.1 dataset [31]. The Strahler stream order ranges from 1 to 9 for Europe and quantifies the branching complexity of streams and rivers within a watershed [30]. Stream size typically increases with Strahler order and has been a reliable proxy across North America [32], and is widely adopted for classifying stream size globally and the European Union (data.europe.eu). We used the “Join Attributes by Location” function in QGIS to link abundance records and the Strahler stream order spatially, i.e., based on the geographical coordinates. For records that could not be directly matched (e.g., due to slight spatial mismatches), we applied a nearest-neighbor join to assign the closest Strahler stream order.

To evaluate the representativeness of the sampling locations with respect to river size, the theoretical Strahler order distribution was calculated for each country from the ECRINS hydrographic dataset. We then evaluated representativeness of river sampling through comparison between the sampled and theoretical distribution.

To link land cover to the abundance records, we assigned the CORINE Land Cover 2018 type (CLC) [33] per sampling location. We used four main categories: urban areas (artificial surfaces including built-up regions such as cities and industrial zones), agricultural land, natural vegetation (forest and semi-natural areas), and wetlands. We matched the abundance records to the surrounding land cover using the “Join Attributes by Location” function in QGIS. When multiple land cover types overlapped geographically with the abundance record, the land cover type with the largest overlapping area around the abundance record was selected.

### Questionnaire

A questionnaire (S1 File) was distributed to all data contributors to gather information on the dataset, determine what part of the data can be publicly shared, and assess challenges limiting data accessibility. The questions covered two aspects: (1) information on the methodology used to collect the data, and (2) information on why and how biodiversity data are shared by each data contributor. We tried to fill missing data by searching literature mentioned by the data contributors.

### FAIR assessment

To ensure that the compiled dataset and metadata are (re)usable by others, we focus on compliance with the FAIR principles, i.e., ensuring that the dataset and accompanying metadata are FAIR – Findable, Accessible, Interoperable, and Reusable. To evaluate the FAIRness of our dataset, we employed the automated tool F-UJI developed by the FAIRsFAIR initiative [34]. This tool systematically measures the extent to which research data objects – including data, metadata, and documentation – adhere to FAIR object assessment principles.

## Results

### Dataset collection

The compiled dataset comprises 2,372,783 macroinvertebrate abundance records, coming from 49 data contributors (see S2 Table) and one national open-access database with a sufficient level of detail [35]. The majority of the macroinvertebrate field data collected was thus obtained through academic researchers who provided scientific data or contacts of data holders of national monitoring, which is rarely publicly available. The European Environment Agency (EEA) WISE platform offers only Ecological Quality Ratio (EQR) values, without the underlying taxa abundance data. The Global Biodiversity Information Facility (GBIF; www.gbif.org) serves as a comprehensive repository of distribution records, encompassing diverse datasets, including some national monitoring data (e.g., Belgium). However, these GBIF macroinvertebrate records often lacked sampling information, and monitoring datasets were sometimes incorporated into larger compilations that prohibited to find specific origins of individual abundance records.

**Fig 1:**
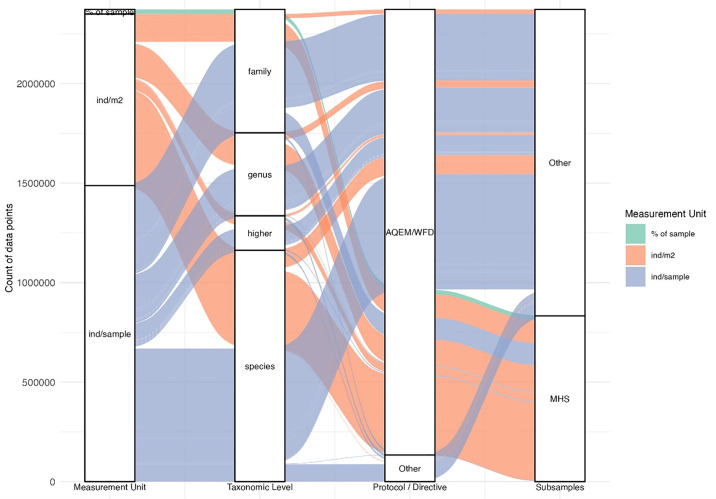
Diversity in the dataset for (1) measurement unit, (2) taxonomic level, (3) protocol or directive followed and (4) the number of subsamples taken.

The dataset exhibits a diversity in measurement units, taxonomic levels, methods, protocols and subsampling techniques (Fig 1). Protocols include those adhering to AQEM/WFD standards as well as regionally specific protocols. Various subsampling techniques are used, with Multihabitat Sampling (MHS) being the most common.

With the current publication, 89% of the abundance records have been made publicly accessible with consent of the data contributors (https://doi.org/10.5281/zenodo.15268354). The FAIR assessment of the compiled dataset available via https://uvaauas.figshare.com/ with the F-UJI tool resulted in an overall 70% FAIRness, including 86% for findability, 67% for accessibility, 75% for interoperability and 60% for reusability.

A total of 46/49 data contributors (94%) completed the survey (S2 File).

### General dataset information

**Fig 2:**
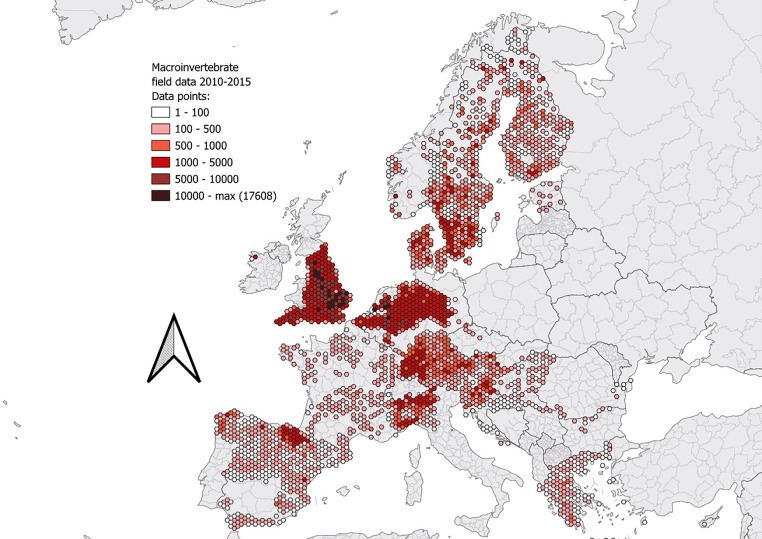
Data density of macroinvertebrate field data from 2010 to 2015. Hexagons represent the number of data points per 30 km grid cell. Base map made with Natural Earth (public domain, naturalearthdata.com).

The compiled dataset contains macroinvertebrate abundance records from 28 countries, including 24 EU Member States (MS) and four non-EU countries (Norway, Serbia, Switzerland, and the United Kingdom). No data were obtained from the following MS: Lithuania, Malta, and Poland, due to data sharing limitations or limited availability. Malta was additionally not included due to its limited relevance for transboundary freshwater analyses. We acknowledge that macroinvertebrate monitoring data exist for countries not represented in our dataset (e.g., [36,37]). 63% of records come from England and Germany, where the highest data density is seen for England and the Netherlands (see Fig 2 and Table 2).

**Table 2:** Number of macroinvertebrate abundance records and data density per country.

*Country*	*Count*	*Data density per km* ^*2*^
*England (UK)*	*1029712*	*7.904*
*Netherlands (NL)*	*142664*	*3.436*
*Luxembourg (LU)*	*4997*	*1.932*
*Belgium (BE)*	*43682*	*1.431*
*Germany (DE)*	*467459*	*1.308*
*Denmark (DK)*	*40797*	*0.947*
*Slovenia (SI)*	*17458*	*0.861*
*Austria (AT)*	*44570*	*0.531*
*Sweden (SE)*	*189110*	*0.420*
*Italy (IT)*	*78342*	*0.260*
*Spain (ES)*	*130043*	*0.257*
*Finland (FI)*	*76966*	*0.228*
*Greece (GR)*	*21408*	*0.162*
*Hungary (HU)*	*12827*	*0.138*
*France (FR)*	*39873*	*0.072*
*Croatia (HR)*	*3529*	*0.062*
*Norway (NO)*	*18607*	*0.058*
*Switzerland (CH)*	*1722*	*0.042*
*Estonia (EE)*	*1418*	*0.031*
*Czech Republic (CZ)*	*1660*	*0.021*
*Ireland (IE)*	*1378*	*0.020*
*Latvia (LV)*	*911*	*0.014*
*Bulgaria (BG)*	*1345*	*0.012*
*Serbia (RS)*	*800*	*0.010*
*Cyprus (CY)*	*76*	*0.008*
*Romania (RO)*	*1097*	*0.005*
*Slovakia (SK)*	*130*	*0.003*
*Portugal (PT)*	*202*	*0.002*

Data density is expressed as the number of records per square kilometer of national land area.

**Fig 3:**
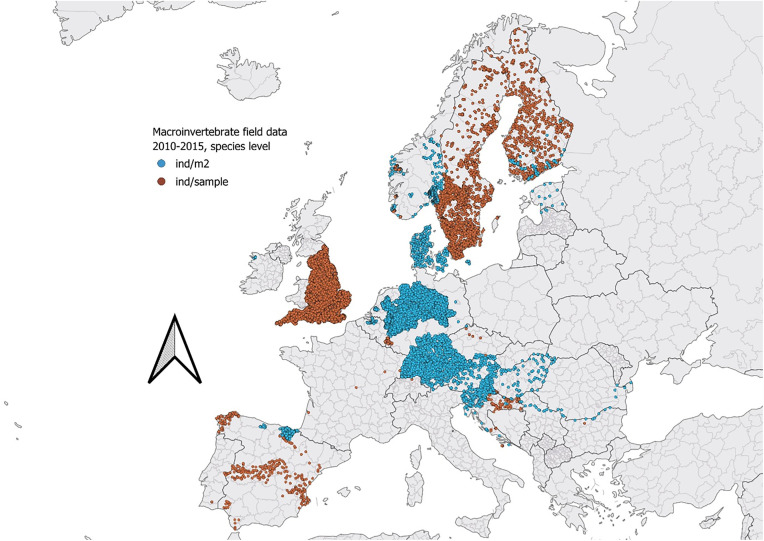
Spatial distribution of species-level macroinvertebrate data across Europe. Data points of macroinvertebrate field data between 2010 and 2015 identified at the species level. Blue: individual counts per metric standard (ind/m²); orange/brown: individual counts per sample. Base map made with Natural Earth (public domain, naturalearthdata.com).

The most common taxonomic level is species, accounting for 49% of the macroinvertebrate abundance records. These species-level observations, representing individual taxa at specific times, are primarily collected in central to northern Europe (see Fig 3). The genus level constitutes 18% of the dataset, while the family level, including subfamily and tribe levels, represents 30% (see S1 and S2 Figs for the spatial distribution at the family and genus levels). Less represented are the class (2%) and order (1%) levels. S3 Table lists the top 20 taxonomic names at the species, genus, and family levels.

Most data (63%) is reported as individual counts per observation or sample, followed by 36% reporting individuals per square meter (m^2^), and 1% reporting individual counts as a percentage of the sample. Fig 3 illustrates the variation in units at the species level.

### Sampling methodology

49% of the taxonomic data was collected for Water Framework Directive (WFD) monitoring and published as EQRs via the European Environment Agency (EEA) WISE database. Additionally, 51% of the dataset consists of a combination of raw WFD monitoring data and research data or supplementary national monitoring data. The vast majority (94%) of the data in the dataset was collected in accordance with protocols aligned with WFD standards, such as the AQEM protocol [38]. Comparable protocols to WFD were also employed, though some research data lacked standardized (inter)national protocols. In over 87% of the sampling locations nets were used, typically hand nets (e.g., Surber nets), and standardized nets for kick-sampling (disturbing the stream bed for a short period), as outlined in the AQEM sampling protocol [39]. Comparable CEN and ISO standards are also commonly used in macroinvertebrate monitoring under the WFD. Sampling protocol variations among countries were made for alignment with national monitoring schemes. These variations include methodological adaptations based on stream size and may include the application of a core sampler, grab sampler, artificial substrates, or a variation in subsamples. For the complete dataset, the diversity of subsamples ranges from none (~41% of abundance records) to as many as 21 subsamples which aligns with the Multi-Habitat Sampling (MHS) scheme. See S4 Table for a list of all 15 subsample categories. In addition, the timing of sampling varies considerably among countries (S5 Table). While many countries focus sampling efforts on spring months (e.g., Denmark, Germany, Netherlands), others sample primarily in autumn (e.g., Finland, Sweden) or throughout the year (e.g., England, Spain, Italy).

On the feasibility to comply with a single, strict EU-wide protocol, the majority of responses indicated significant challenges and reservations. 39% of respondents stated that such adjustments would not be feasible due to various constraints, including national policies and potential impacts on long-term trend analyses. Conversely, 33% of respondents expressed willingness to adjust or align with other protocols. Meanwhile, 28% were uncertain, suggesting that alignment might be possible under specific conditions, provided a close alignment with existing protocols.

### Strahler stream order distribution and land cover

**Fig 4:**
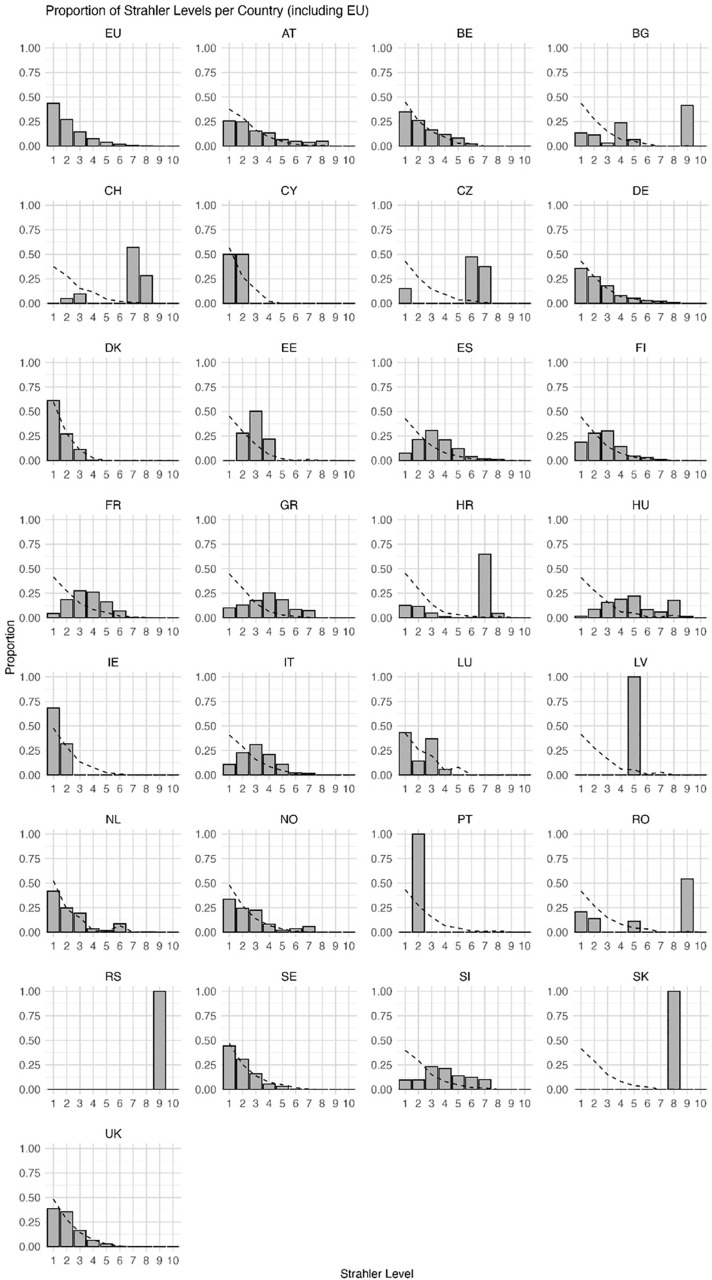
Strahler order distribution of macroinvertebrate sampling locations per country. Bars represent the distribution of Strahler orders in the dataset. The dashed black line represents the theoretical Strahler order distribution of rivers within each country.

The total Strahler stream order distribution of the locations with macroinvertebrate abundance records did not fully align with the Strahler stream order distribution in the countries (Fig 4). There was a 10% lower representation for small streams (low Strahler stream order) while data for large rivers were overrepresented. For countries with a large contribution to the dataset (UK and Germany), the Strahler stream order distribution is well represented. In the data obtained for this study, several countries (e.g., Portugal, Switzerland, and Slovakia) show a limited representation of stream orders and smaller streams (orders 1 and 2) tend to be underrepresented, though more comprehensive monitoring data may exist at the national level (e.g., [40]).

**Fig 5:**
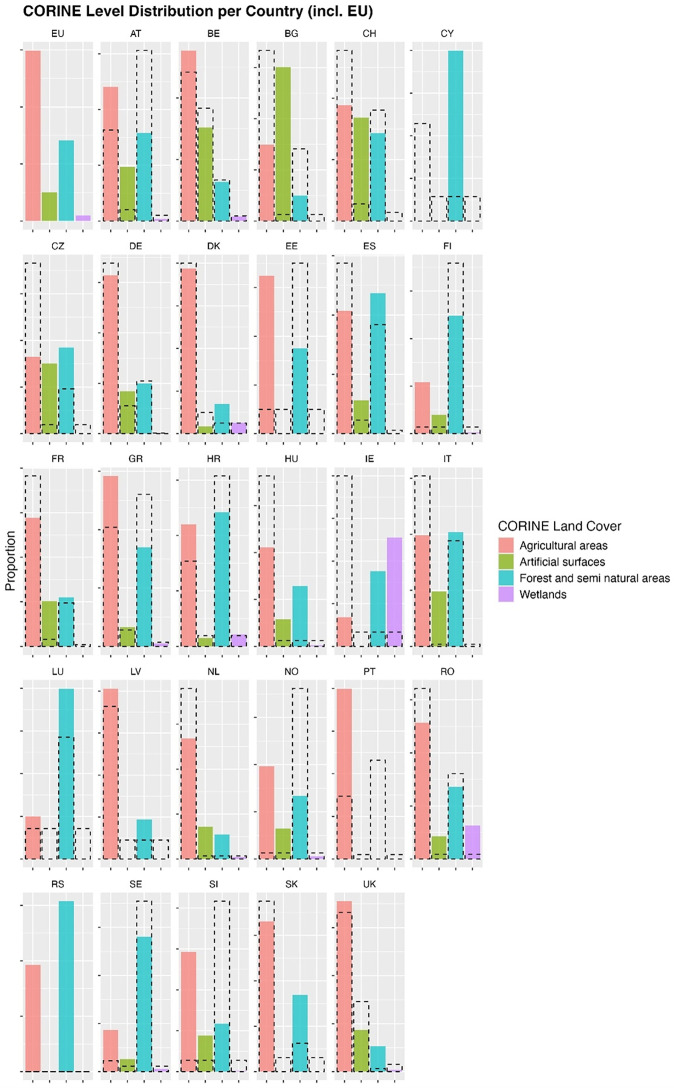
Land cover representation of macroinvertebrate sampling locations per country. Bars represent the CORINE Land Cover (CLC) 2018 distribution in the dataset per country, with the aggregated EU distribution shown as a separate facet. The dashed black line represents the CLC land cover distribution in river basins within each country.

Macroinvertebrate abundance records from river catchments dominated by urban land use are overrepresented in the dataset, while forest and semi-natural land cover are slightly underrepresented (Table 3). A comparable analysis per country shows that over- or underrepresentation of land cover is severe for most of the contributing countries (Fig 5).

**Table 3:** Dominating CLC land cover of data points and EU rivers.

*CLC land use*	*Land cover of data points (%)*	*Land cover of rivers of the countries included in the dataset (%)*
*Agricultural areas*	*59.6*	*61.2*
*Artificial surfaces*	*15.9*	*9.7*
*Forest and semi natural areas*	*23.7*	*28.6*
*Wetlands*	*0.9*	*0.4*

### Data accessibility according to data contributors

The questionnaire showed that data sharing was not facilitated by the work environment of 37% of the data contributors. Conversely, 57% reported that data sharing was facilitated with a proactive approach with public repositories, institutional support, training initiatives, and integrated work processes. Data contributors are generally willing to share data when they trust that the data will be used with care, for example when the aim is a high impact journal, or when they are co-author or acknowledged in scientific papers. For governmental institutions the policy is often that data should be shared when no sensitive information is included (e.g., the location of vulnerable species). Amongst the data contributors there is substantial support for using standardized templates, to enhance data accessibility and meet regulatory requirements. However, practices still significantly vary. Many data provided to the compiled dataset in this study is already available, e.g., via international databases (11%), data repositories or supplementary materials (20%) linked to, e.g., scientific journals or specific river systems, or via national databases (26%). However, a large part of our dataset was only provided after direct contacting the data contributor (43%).

Suggestions by the data contributors to adjust the data sharing system include (1) standardizing data storage and format guidelines, (2) establishing central national and European repositories, (3) offering co-authorship or career recognition through repositories with impact factor and citation as incentives, (4) securing funding for data management and collection, (5) enforcing data sharing through European regulations and contractual obligations and (6) ensuring data quality through a central organ with gate-keeper capacity and data sharing requirements.

## Discussion

In this work we compiled raw data of macroinvertebrate abundances for rivers and lakes across Europe resulting in over 2.3 million records for the first WFD cycle (a 6-year period from 2010 to 2015). Despite similarities in protocols at national level, transnational comparisons on a European scale constrain comparability due to the divergent methods being employed.

### Variability in methodology

To address the Essential Biodiversity Variable (EBV) ‘Community composition of benthic invertebrates’ (relative) abundances of benthic invertebrates are preferred [41]. In our dataset only 49% of the records is abundance data at species level. To increase species specific information across Europe, it is therefore recommended that many national monitoring programs transition toward collecting data at species level where possible. While we recommend that national monitoring programs transition toward species-level identification where possible, we acknowledge that this remains challenging, particularly for diverse groups, where traditional morphological identification is time-consuming and requires specialized expertise.

Standardized identification keys and training programs could help address this challenge [42]. The development of an EU-defined standardized operational taxonomic list would be an important step toward reducing taxonomic inconsistencies across national and regional scales, as the WFD currently lacks explicit taxonomic requirements for biological quality elements. While the AQEM/STAR taxa list [38] provides a valuable framework, it was originally developed based on western and central European fauna and may not fully represent the taxonomic diversity across all European regions. DNA-based identification methods could be used as a complement to traditional taxonomy to help address this challenge [43].

Discrepancies in the compiled dataset exist concerning the unit (individual counts per metric standard or per sample) and the number of subsamples used. Both issues can be standardized, for example following the AQEM sampling method [44]. However, adoption of the AQEM method allows flexibility to tailor sampling and monitoring techniques, provided they align with the overarching principles of the WFD. As a result, methodologies are comparable per member state, but between member states methods vary importantly. Species abundance data converted to abundance classes allow for comparison across countries [45]. Furthermore, the timing of sampling varies among countries, which can influence community composition due to seasonal variation in macroinvertebrate life cycles and represents an additional source of variation in transnational comparisons.

A quarter of a century after the adoption of the WFD, possibilities for further standardizing data collection are well-known but still remain challenging, as also the questionnaire responses show. Many data holders exhibit reluctance to modify their programs or would agree to changes only under specific conditions, because a transition to a more standardized EU-wide protocol can have potential impacts on the continuity of existing long-term monitoring programs and as a result complicating long-term trend analyses. To facilitate transnational comparisons on a European scale using Ecological Quality Ratios (EQRs) individual water bodies have been aggregated [22] and these indicator values can be useful for trend analyses on a European scale. However, to transnationally assess the effect of stressors on biodiversity on a finer scale, further standardization is encouraged.

### Data representativeness

The spatial distribution or available data density can be strongly improved for many European countries. However, despite all our labor-intensive efforts we cannot exclude that additional data still are available. The currently limited representativeness is, at least in part, a result of the risk-based design of many national monitoring programs, which tend to focus on larger rivers due to their exposure to known pressures [13]. As a result, smaller and/or intermittent rivers—despite their known contribution to biodiversity [46–49]—remain underrepresented. In some countries, like England, this challenge is being addressed by setting up complementary surveillance networks using spatially balanced sampling designs [50]. Moreover, improving land-use representativeness remains important, as land use is a key factor shaping biodiversity patterns [51,52].

### Data sharing and data access

The FAIR principles provide guidelines to ensure that data are shared in a way that maximizes their value and usability [53], with the recognition that digital resources should be capable of accessing the data publication autonomously. The aim of this work is to increase the FAIRness of the collected data by making quality-checked aquatic macroinvertebrate abundance records available through an assigned persistent identifier (https://doi.org/10.5281/zenodo.15268354). The data are shared under the CC BY 4.0 license and are provided in standardized machine-readable format, to further enhance biodiversity research. Quality assurance of the data was primarily ensured through direct contact with the data contributors, as reflected in the questionnaire responses.

This highlights the importance of creating robust data-sharing infrastructures, which would be beneficial for both data holder and data user and initiatives such as the proposed EU Biodiversity Observation Coordination Centre (EBOCC) [54]. An investment into an EBOCC would improve coordination for biodiversity monitoring in Europe, including (1) data management (data mobilization, integration and harmonization, improved sampling designs and standardization of data collection, data infrastructure and tools, development of data access and data sharing policies), (2) coordination (support for coordination between Member States and institutions, international coordination), and (3) capacity building (e.g., support for data exchange, analysis and standardization). The existing EEA WISE platform, a centralized platform for the WFD biological data, could be enhanced to facilitate raw biology data integration, validation and storage, including comprehensive information on the methodologies employed for field data collection. Another widely recognized data aggregation infrastructure is the Global Biodiversity Information Facility (GBIF), which could be improved to incorporate mandatory methodology and data validation parameters (e.g., using specific guidelines, the number of subsamples and the sampling unit), allowing it to serve as a robust platform for comprehensive biodiversity data management and sharing.

A centralized platform might foster research beyond the scope of the WFD and EQRs, for example research on anthropogenic causes of biodiversity loss in European freshwater ecosystems.

## Concluding remarks

Our study focuses on the availability and applicability of raw macroinvertebrate field data across European water bodies. The compiled dataset of over 2.3 million abundance records revealed methodological inconsistencies.

Spatial coverage does not represent the whole of the continent evenly, with major gaps in eastern and southern Europe, and underrepresentation of smaller streams and certain land-uses. Methodological diversity, including varying measurement units and taxonomic resolutions, is a challenge for transnational comparisons. To improve data sharing practices, institutional barriers and individual incentives should be addressed, next to standardizing data formats, and ensuring robust quality control mechanisms. This study underscores the need for a centralized platform to facilitate data validation and sharing, with initiatives such as EBOCC, WISE and GBIF offering potential solutions. Further standardization, harmonization and integration can boost biodiversity research and the understanding and mitigation of biodiversity loss in freshwater ecosystems. Such knowledge is needed to meet the EU Biodiversity strategy and restoration law. Addressing these challenges is crucial for advancing research on biodiversity loss and environmental management in freshwater ecosystems across Europe.

## Supporting information

S1 Table Overview of national and international databases consulted for relevant biological data. (DOCX)

S1 File Questionnaire sent to data contributors on dataset content and accessibility. (DOCX)

S2 Table Data contributors and dataset sources. (DOCX)

S1 Fig Spatial distribution of family-level macroinvertebrate data across Europe. Data points of macroinvertebrate field data between 2010 and 2015 identified at the family level. Base map made with Natural Earth (public domain, naturalearthdata.com).
(TIFF)

S2 Fig Spatial distribution of genus-level macroinvertebrate data across Europe. Data points of macroinvertebrate field data between 2010 and 2015 identified at the genus level. Base map made with Natural Earth (public domain, naturalearthdata.com).
(TIFF)

S3 Table Top 20 taxonomic names of the taxonomy levels species, genus, and family of the dataset. The percentage of data indicates the frequency of the taxonomic name in the dataset.
(DOCX)

S4 Table Diversity in the number of subsamples within the dataset. Including the percentage of the dataset’s data points with that specific number of subsamples.
(DOCX)

S5 Table Number of macroinvertebrate abundance records (n) per country and sampling month (1–12). (DOCX)

S2 File Answers to the questionnaire Part 2 and Part 3. (DOCX)
