# Supplementary material for: Availability and representativeness of freshwater macroinvertebrate data to enable Europe-wide assessment of biodiversity trends
Source: PLoS One. 2026 Aug 13;21(8):e0354723. doi: 10.1371/journal.pone.0354723 (PMC13472404; doi:10.1371/journal.pone.0354723)
Supplement: Supplementary file 2 [file pone.0354723.s001.docx]

**S1 Table. Overview of national and international databases consulted for relevant biological data.**

| **Database Name** | **URL** | **Description** |
| --- | --- | --- |
| **European & International Biodiversity Networks** | | |
| ITIS (Integrated Taxonomic Information System) | [https://www.itis.gov](https://www.itis.gov/) | Standardized taxonomic database for species classification. |
| DEIMS-SDR | [https://deims.org](https://deims.org/) | Database for ecological research sites, including Long-Term Ecological Research (LTER) locations. |
| DataONE | [https://search.dataone.org](https://search.dataone.org/) | Scientific data repository. |
| EUDAT B2Find | [https://b2find.eudat.eu](https://b2find.eudat.eu/) | European search engine for scientific data. |
| GBIF (Global Biodiversity Information Facility) | [https://www.gbif.org](https://www.gbif.org/) | Global database of biodiversity occurrences and species records. |
| LifeWatch | <https://www.lifewatch.be/data-services/> | European infrastructure for biodiversity and ecosystem research. |
| EMODnet Biology | <https://www.emodnet-biology.eu/portal/> | Marine biodiversity data portal for Europe. |
| Biodiversity Data Journal (BDJ) | [https://bdj.pensoft.net](https://bdj.pensoft.net/) | Open-access journal for biodiversity data publication. |
| ICPR (International Commission for the Protection of the Rhine) | <https://www.iksr.org/en/> | Organization focused on the protection and monitoring of the Rhine River. |
| ICPDR (International Commission for the Protection of the Danube River) | [https://www.icpdr.org](https://www.icpdr.org/) | Research and monitoring data on the Danube River. |
| ICPER (International Commission for the Protection of the Elbe) | <https://www.ikse-mkol.org/en> | Elbe River protection. |
| Biodiversity Europe | <https://biodiversity.europa.eu/ecosystems/rivers-and-lakes> | European biodiversity portal. |
| eLTER Europe | <https://lter-europe.net/> | Long-Term Ecosystem Research network for European biodiversity and environmental monitoring. |
| **Country-Specific Databases** | | |
| Austria - Bioportal Austria | [https://biocache.biodiversityatlas.at](https://biocache.biodiversityatlas.at/) | Austrian biodiversity data. |
| Austria - UBA Water Information System | <https://wasser.umweltbundesamt.at/wisa-datenabfrage> | Water quality and monitoring data. |
| Belgium - INBO | <https://www.vlaanderen.be/inbo> | Belgian biodiversity monitoring. |
| Croatia - Bioportal | [http://www.haop.hr](http://www.haop.hr/) | Biodiversity data in Croatia. |
| Croatia - Voda.hr | [https://www.voda.hr](https://www.voda.hr/) | Croatian water resources data. |
| Denmark - NERI | <https://ecos.au.dk/forskningraadgivning/fagdatacentre/ferskvand> | Freshwater monitoring in Denmark. |
| Estonia - Ministry of Environment | <https://www.envir.ee/en> | Environmental monitoring in Estonia. |
| Finland - Laji.fi | <https://laji.fi/> | Finnish biodiversity monitoring. |
| France - Naiades | [http://www.naiades.eaufrance.fr](http://www.naiades.eaufrance.fr/) | National water monitoring data. |
| France - Open Data France | <https://support.data.gouv.fr/merci> | Government open data portal. |
| Germany - BfN | [https://www.bfn.de](https://www.bfn.de/) | Nature conservation in Germany. |
| Germany - GLD Sachsen-Anhalt | <https://gld.lhw-sachsen-anhalt.de/> | Regional environmental data. |
| Germany - GFBio | <https://www.gfbio.org> | Data infrastructure for managing and archiving biological and environmental research data in Germany |
| Hungary - Vadonleső | <https://vadonleso.hu/> | Hungarian biodiversity monitoring. |
| Hungary - Nature Protection Database | [https://termeszetvedelem.hu](https://termeszetvedelem.hu/) | Hungarian nature conservation. |
| Ireland - Biodiversity Ireland | [https://biodiversityireland.ie](https://biodiversityireland.ie/) | Irish biodiversity data. |
| Italy - ISPRA | [https://www.isprambiente.gov.it](https://www.isprambiente.gov.it/) | Italian environmental data. |
| Latvia - Nature Data Portal | <https://latvianature.daba.gov.lv/en/> | Latvian biodiversity monitoring. |
| Lithuania - Environmental Monitoring | [https://am.lrv.lt](https://am.lrv.lt/) | National environmental monitoring. |
| Luxembourg - LIST | [https://www.list.lu](https://www.list.lu/) | Climate and biodiversity monitoring. |
| Poland - GIOS | [https://www.gios.gov.pl](https://www.gios.gov.pl/) | Environmental monitoring in Poland. |
| Romania - INCDPM | [http://www.incdpm.ro](http://www.incdpm.ro/) | Environmental research in Romania. |
| Slovakia - Enviroportal | [https://www.enviroportal.sk](https://www.enviroportal.sk/) | Slovak environmental monitoring. |
| Slovenia - ARSO | [https://www.arso.gov.si](https://www.arso.gov.si/) | Environmental indicators. |
| Sweden - Miljödata SLU | <https://miljodata.slu.se/MVM> | Swedish environmental data. |
| Sweden - Artportalen | [https://artportalen.se](https://artportalen.se/) | Swedish biodiversity database. |
| UK - Environment Agency Biodiversity Data | <https://environment.data.gov.uk/ecology/explorer> | Biodiversity monitoring in the UK. |
