# Supplementary material for: Availability and representativeness of freshwater macroinvertebrate data to enable Europe-wide assessment of biodiversity trends
Source: PLoS One. 2026 Aug 13;21(8):e0354723. doi: 10.1371/journal.pone.0354723 (PMC13472404; doi:10.1371/journal.pone.0354723)
Supplement: Supplementary file 3 [file pone.0354723.s002.docx]

**S1 File. Questionnaire sent to data contributors on dataset content and accessibility.**

The questionnaire consists of three parts:

1. **Data contributor** – contact information of the data holder

2. **Biodiversity data** – information on the methodology

3. **Data sharing** – information on why and how biodiversity data are being shared

**PART 1: Data contributor**

Contact information of the data holder

1.	Name

2.	Email

3.	Country (optional area)

4.	Would you like to be a co-author of the manuscript?

a.	Yes

b.	No

c.	Only after contribution to the manuscript

**PART 2: Biodiversity data**

Information on the methodology

1.	Does the monitoring data consist of raw Water Framework Directive (WFD) data? Yes/No/Partly

2.	Was the fieldwork conducted following a WFD protocol?

a.	Yes, the protocol used is according to the WFD, but was adjusted to the existing monitoring scheme in my country

b.	No, a different kind of protocol was used

c.	Other:

3.	Was sampling performed differently depending on the size of the stream?

a.	Yes

b.	No

4.	The sampling was performed differently depending on the size of the stream. Please elaborate (e.g. a small stream was considered ..): …

5.	Was the fieldwork done using a hand net or a core sampler or both depending on the field situation?

a.	A hand net resulting in individual count per sample

b.	A core sampler resulting in individual count per metric standard (e.g. m^2^)

c.	Both were used, resulting in individuals per metric standard as well as individuals per sample

d.	Other: …

6.	Does one sample consist of an average of multiple subsamples?

a.	Yes, according to the multihabitat sampling (MHS) scheme

b.	Yes

c.	No

d.	Other:

7.	What is the number of subsamples per sample? If this number varies, please describe why.

8.	Would it be possible to adjust your field work and lab work approach for one specific EU wide strict protocol (i.e. determination at one specific taxon level, using one specific methodology like core sampler) in order to make data more comparable on a regional scale? Please elaborate.

9.	Can I make the macroinvertebrate data that you shared with me publicly available in .xlsx form after publishing? I will send the final sheet form for approvement.

a.	Yes

b.	Yes, but only after a certain time - Please specify the time frame after which the data can be made available

c.	No - Please elaborate why the data cannot become available

**PART 3: Data sharing**

information on why and how biodiversity data is being shared

1.	Are you familiar with the FAIR (Findable, Accessible, Interoperable, and Reusable) data principles?

a.	Yes

b.	No

c.	Other:

2.	Is data sharing being facilitated within your work environment through for example data management training, funds and/or time?

a.	Yes

b.	No, but we are expected to do it in our own time

c.	No, nothing is expected of me to share any data

d.	Other

3.	How is data sharing being facilitated?

4.	Are you encouraged to archive the data using a standardised template (e.g. a defined spreadsheet like the Darwin Core template from gbif)?

a.	Yes, to help create accessible data for the public

b.	Yes, but only for the Water Framework Directive (WFD)

c.	No, I use my own approach

d.	Other

5.	How is your data available (if multiple, please use 'other' and describe the two or more options):

a.	By contacting me directly

b.	Via a national database

c.	Via an international database

d.	Via a data repository

e.	Other

6.	What kind of data are available when present in a database or data repository?

a.	All raw data, including data of less quality (e.g. data that are not checked)

b.	Only raw data that is checked and of sufficient quality

c.	Adjusted data, for example only ecological status data or occurrence data

d.	I don’t have data available in a database

7.	When you are hesitant to share data, how would you become more encouraged? Multiple answers are possible:

a.	When I trust the data is used with care

b.	When the person asking contains a high profile (e.g. a professor)

c.	When I will be credited as co-author of a scientific article

d.	When I will be acknowledged in the acknowledgements of a scientific article

e.	I do not have an opinion about this because our work policy is to always share data

f.	Other

**PART 3: Data sharing** *(complaints and improvements)*

With biodiversity being a very hot topic, sharing of monitoring data is encouraged. For example, funds are only given when data are shared at the end of the project, or journals will only publish manuscripts when data are made available. However, there seems to be a gap between policy, research and practice.

1.	Do you have any complaints on how the system on sharing data is currently being executed:

2.	Would you have any suggestions on how to change the data sharing system through e.g. time, funds, education, co-authorship, etc.? What do you think is needed for you as a data holder?

Do you have any last comments about the questionnaire or other matters related to the content of the manuscript on availability of biodiversity data in Europe?
