# Supplementary material for: Availability and representativeness of freshwater macroinvertebrate data to enable Europe-wide assessment of biodiversity trends
Source: PLoS One. 2026 Aug 13;21(8):e0354723. doi: 10.1371/journal.pone.0354723 (PMC13472404; doi:10.1371/journal.pone.0354723)
Supplement: Supplementary file 4 [file pone.0354723.s003.docx]

**S2 Table. Data contributors and dataset sources.**

| **Data provider** | **Number of data points** | **Percentage of total** | **Country of data points** | **Academic or governmental institution** |
| --- | --- | --- | --- | --- |
| 1 | 913700 | 38,51 | England | UK Environment Agency |
| 2 | 167726 | 7,07 | Sweden | Miljödata-MVM, Swedish University of Agricultural Science (SLU) (Sweden) |
| 3 | 142664 | 6,01 | Netherlands | Wageningen University & Research (Netherlands) |
| 4 | 141775 | 5,97 | Germany | State Agency for Nature, Environment and Consumer Protection North Rhine-Westphalia (LANUV), Germany |
| 5 | 112210 | 4,73 | England | UK Environment Agency |
| 6 | 103163 | 4,35 | Germany | Lower Saxony Water Management, Coastal Defence and Nature Conservation Agency (NLWKN), Germany |
| 7 | 97177 | 4,10 | Spain | University of Valencia (Spain) |
| 8 | 77156 | 3,25 | Italy | University of Milano-Bicocca and Water Research Institute IRSA (Italy) |
| 9 | 76837 | 3,24 | Germany | State Office for Environment, Agriculture and Geology of Saxony (LHW Sachsen), Germany |
| 10 | 75179 | 3,17 | Finland | Finnish Environment Institute (SYKE) |
| 11 | 62805 | 2,65 | Germany | State Institute for the Environment Baden-Württemberg (LUBW), Germany |
| 12 | 51291 | 2,16 | Germany | Bavarian State Office for the Environment (LfU), Germany |
| 13 | 43488 | 1,83 | Austria | Federal Ministry of Agriculture, Regions and Tourism (BML), Austria |
| 14 | 41338 | 1,74 | Belgium | Flanders Environment Agency (VMM), Belgium |
| 15 | 40797 | 1,72 | Denmark | Norwegian Institute for Water Research (NIVA), Denmark |
| 16 | 39794 | 1,68 | France | University of Lyon (UDL), France |
| 17 | 25266 | 1,06 | Spain | University of the Basque Country (UPV/EHU), Spain |
| 18 | 23300 | 0,98 | Germany | Saxon State Office for Environment, Agriculture and Geology (LfULG), Germany |
| 19 | 21408 | 0,90 | Greece | Hellenic Centre for Marine Research (HCMR), Greece |
| 20 | 17458 | 0,74 | Slovenia | Ministry of Environment, Climate and Energy (Slovenia) |
| 21 | 14121 | 0,60 | Norway | Norwegian Institute for Water Research (NIVA), Norway |
| 22 | 12241 | 0,52 | Sweden | Swedish University of Agricultural Sciences (SLU), Sweden |
| 23 | 11368 | 0,48 | Hungary | Department of Hydrobiology, University of Pécs, Hungary |
| 24 | 9143 | 0,39 | Sweden | Swedish University of Agricultural Sciences (SLU), Sweden |
| 25 | 6152 | 0,26 | Germany, Slovakia, Hungary, Serbia, Romania, Bulgaria, Austria | International Commission for the Protection of the Danube River (ICPDR) |
| 26 | 5562 | 0,23 | Spain | University of Barcelona (UB), Spain |
| 27 | 4997 | 0,21 | Luxembourg | Luxembourg Institute of Science and Technology (LIST), Luxembourg |
| 28 | 4486 | 0,19 | Norway | NORCE Norwegian Research Centre AS, Norway |
| 29 | 3802 | 0,16 | England | UK Environment Agency |
| 30 | 3779 | 0,16 | Germany | Elbe River Basin Community (FGG Elbe), Germany |
| 31 | 3529 | 0,15 | Croatia | Ministry of Economy and Sustainable Development, Croatia |
| 32 | 2439 | 0,10 | France, Switzerland, Germany, Austria | International Commission for the Protection of the Rhine (ICPR) |
| 33 | 2344 | 0,10 | Belgium | Research Institute for Nature and Forest (INBO), Belgium |
| 34 | 2166 | 0,09 | Spain | Environmental Hydraulics Institute of the University of Cantabria (IHCantabria), Spain |
| 35 | 1787 | 0,08 | Finland | University of Oulu, Finland |
| 36 | 1418 | 0,06 | Estonia | Estonian University of Life Sciences (EMU), Estonia |
| 37 | 1410 | 0,06 | Czech Republic | Czech Hydrometeorological Institute, Czech Republic |
| 38 | 1378 | 0,06 | Ireland | Marine Institute, Ireland |
| 39 | 1279 | 0,05 | Germany | University of Duisburg-Essen, Germany |
| 40 | 1034 | 0,04 | Italy | APEM Group, Italy |
| 41 | 911 | 0,04 | Latvia | University of Latvia, Latvia |
| 42 | 837 | 0,04 | Germany | Technical University of Kaiserslautern-Landau, Germany |
| 43 | 768 | 0,03 | Germany | Senckenberg Research Institute and Natural History Museum, Germany |
| 44 | 365 | 0,02 | Bulgaria | Institute of Biodiversity and Ecosystem Research, Bulgarian Academy of Sciences, Bulgaria |
| 45 | 314 | 0,01 | Austria | University of Natural Resources and Life Sciences (BOKU), Austria |
| 46 | 250 | 0,01 | Czech Republic | Masaryk University, Brno, Czech Republic |
| 47 | 202 | 0,01 | Portugal | University of Coimbra & Marine and Environmental Sciences Centre, Portugal |
| 48 | 152 | 0,01 | Italy | University of Milano-Bicocca, Italy |
| 49 | 86 | 0,00 | Switzerland | University of Zurich, Switzerland |
| 50 | 76 | 0,003 | Cyprus | Water Development Department, Ministry of Agriculture, Rural Development and Environment, Cyprus |
