# Supplementary material for: Availability and representativeness of freshwater macroinvertebrate data to enable Europe-wide assessment of biodiversity trends
Source: PLoS One. 2026 Aug 13;21(8):e0354723. doi: 10.1371/journal.pone.0354723 (PMC13472404; doi:10.1371/journal.pone.0354723)
Supplement: Supplementary file 5 [file pone.0354723.s006.docx]

**S3 Table. Top 20 taxonomic names of the taxonomy levels species, genus, and family of the dataset.** The percentage of data indicates the frequency of the taxonomic name in the dataset.

| **Species** | **% of total data** |  | **Genus** | **% of total data** |  | **Family** | **% of total data** |
| --- | --- | --- | --- | --- | --- | --- | --- |
| *Asellus aquaticus* | 2.8 |  | *Baetis sp.* | 5.4 |  | Chironomidae gen. sp. | 11.3 |
| *Elmist aenea* | 2.6 |  | *Hydropsyche sp.* | 3.9 |  | Baetidae gen. sp. | 4.6 |
| *Baetis rhodani* | 2.5 |  | *Bisidium sp.* | 2.9 |  | Limnephilidae gen. sp. | 4.5 |
| *Hydropsyche siltalai* | 2.1 |  | *Rhyacophila sp.* | 2.6 |  | Elmidae gen. sp. | 4.5 |
| *Limnius volckmari* | 1.9 |  | *Leuctra sp.* | 2.4 |  | Hydropsychidae gen. sp. | 3.1 |
| *Polycentropus flavomaculatus ssp.* | 1.8 |  | *Elmis sp.* | 2.1 |  | Sphaeriidae gen. sp. | 2.8 |
| *Rhyacophila nubila* | 1.5 |  | *Oulimnius sp.* | 1.9 |  | Gammaridae gen. sp. | 2.8 |
| *Hydropsyche pellucidula* | 1.5 |  | *Asellus sp.* | 1.9 |  | Simuliidae gen. sp. | 2.7 |
| *Erpobdella octoculata* | 1.5 |  | *Polycentropus sp.* | 1.9 |  | Planorbidae gen. sp. | 2.1 |
| *Lepidostoma hirtum* | 1.4 |  | *Nemoura sp.* | 1.8 |  | Tubificidae gen. sp. | 2.1 |
| *Sericostoma personatum* | 1.4 |  | *Gammarus sp.* | 1.7 |  | Leptoceridae gen. sp. | 2.1 |
| *Baetis niger* | 1.3 |  | *Isoperla sp.* | 1.7 |  | Heptageniidae gen. sp. | 2.1 |
| *Ancylus fluviatilis* | 1.3 |  | *Caenis sp.* | 1.6 |  | Nemouridae gen. sp. | 2.1 |
| *Leuctra hippopus* | 1.3 |  | *Amphinemura sp.* | 1.6 |  | Glossiphoniidae gen. sp. | 1.9 |
| *Heptagenia sulphurea* | 1.3 |  | *Limnius sp.* | 1.6 |  | Erpobdellidae gen. sp. | 1.8 |
| *Potamopyrgus antipodarum* | 1.2 |  | *Leptophlebia sp.* | 1.5 |  | Polycentropodidae gen. sp. | 1.7 |
| *Protonemura meyeri* | 1.1 |  | *Dicranota sp.* | 1.4 |  | Asellidae gen. sp. | 1.7 |
| *Oulimnius tuberculatus* | 1.1 |  | *Erpobdella sp.* | 1.3 |  | Rhyacophilidae gen. sp. | 1.7 |
| *Glossiphonia complanata* | 1.1 |  | *Heptagenia sp.* | 1.2 |  | Dytiscidae gen. sp. | 1.6 |
| *Caenis luctuosa* | 1.1 |  | *Hydraena sp.* | 1.2 |  | Ceratopogonidae gen. sp. | 1.4 |
