# Supplementary material for: Availability and representativeness of freshwater macroinvertebrate data to enable Europe-wide assessment of biodiversity trends
Source: PLoS One. 2026 Aug 13;21(8):e0354723. doi: 10.1371/journal.pone.0354723 (PMC13472404; doi:10.1371/journal.pone.0354723)
Supplement: Supplementary file 6 [file pone.0354723.s007.docx]

**S4 Table. Diversity in the number of subsamples within the dataset.** Including the percentage of the dataset's data points with that specific number of subsamples.

| ***number of subsamples*** | ***data points (%)*** |  |  |
| --- | --- | --- | --- |
|  |  |  |  |
| *0* | *41.1* |  |  |
| *1* | *6.3* |  |  |
| *2* | *1.8* |  |  |
| *3* | *0.7* |  |  |
| *4* | *0.3* |  |  |
| *5* | *7.1* |  |  |
| *6* | *0.2* |  |  |
| *7* | *<0.1* |  |  |
| *8* | *<0.1* |  |  |
| *9* | *0.1* |  |  |
| *10* | *0.3* |  |  |
| *12* | *1.9* |  |  |
| *depends on site (habitat and occurrence)* | *5.1* |  |  |
| *MHS* | *35.1* |  |  |
| *unknown* | *<0.1* |  |  |
