# Supplementary material for: Availability and representativeness of freshwater macroinvertebrate data to enable Europe-wide assessment of biodiversity trends
Source: PLoS One. 2026 Aug 13;21(8):e0354723. doi: 10.1371/journal.pone.0354723 (PMC13472404; doi:10.1371/journal.pone.0354723)
Supplement: Supplementary file 7 [file pone.0354723.s008.docx]

**S5 Table. Number of macroinvertebrate abundance records (n) per country and sampling month (1 to 12).**

|  | Month | | | | | | | | | | | |
| --- | --- | --- | --- | --- | --- | --- | --- | --- | --- | --- | --- | --- |
| Country | 1 | 2 | 3 | 4 | 5 | 6 | 7 | 8 | 9 | 10 | 11 | 12 |
| Austria | 1246 | 5603 | 7007 | 2573 | 2495 | 1071 | 6766 | 6470 | 5538 | 3277 | 2524 | 0 |
| Belgium | 0 | 52 | 1958 | 3254 | 4592 | 6469 | 3432 | 3118 | 8153 | 11137 | 1023 | 494 |
| Bulgaria | 0 | 0 | 0 | 0 | 0 | 78 | 58 | 213 | 980 | 16 | 0 | 0 |
| Croatia | 0 | 0 | 20 | 521 | 535 | 724 | 369 | 331 | 397 | 348 | 268 | 16 |
| Cyprus | 0 | 0 | 0 | 76 | 0 | 0 | 0 | 0 | 0 | 0 | 0 | 0 |
| Czech Republic | 0 | 0 | 46 | 663 | 106 | 244 | 46 | 15 | 540 | 0 | 0 | 0 |
| Denmark | 0 | 2167 | 14176 | 23986 | 468 | 0 | 0 | 0 | 0 | 0 | 0 | 0 |
| England | 15563 | 10411 | 134278 | 186307 | 155062 | 12487 | 26591 | 23004 | 190625 | 172299 | 92365 | 10720 |
| Estonia | 0 | 0 | 0 | 95 | 1176 | 0 | 0 | 0 | 147 | 0 | 0 | 0 |
| Finland | 0 | 0 | 0 | 0 | 160 | 0 | 145 | 1509 | 44051 | 23684 | 7133 | 284 |
| France | 0 | 908 | 140 | 0 | 114 | 4819 | 13170 | 14574 | 5594 | 554 | 0 | 0 |
| Germany | 1156 | 2833 | 58789 | 162514 | 114039 | 61845 | 36431 | 15375 | 5632 | 5514 | 3065 | 266 |
| Greece | 0 | 0 | 356 | 7919 | 1164 | 99 | 6486 | 2081 | 2500 | 724 | 32 | 0 |
| Hungary | 0 | 0 | 84 | 2276 | 1886 | 334 | 506 | 2785 | 3191 | 1489 | 276 | 0 |
| Ireland | 0 | 0 | 0 | 30 | 858 | 490 | 0 | 0 | 0 | 0 | 0 | 0 |
| Italy | 1294 | 2558 | 9904 | 6565 | 6915 | 8662 | 10322 | 4709 | 9297 | 11463 | 4292 | 2361 |
| Latvia | 0 | 0 | 0 | 0 | 0 | 0 | 911 | 0 | 0 | 0 | 0 | 0 |
| Luxembourg | 0 | 0 | 0 | 0 | 0 | 0 | 0 | 1959 | 1617 | 1421 | 0 | 0 |
| Netherlands | 398 | 200 | 3219 | 36289 | 49106 | 27279 | 5765 | 1822 | 14222 | 3866 | 177 | 321 |
| Norway | 160 | 0 | 584 | 2368 | 823 | 249 | 0 | 0 | 1451 | 6537 | 6154 | 281 |
| Portugal | 0 | 0 | 0 | 202 | 0 | 0 | 0 | 0 | 0 | 0 | 0 | 0 |
| Romania | 0 | 0 | 0 | 0 | 0 | 0 | 0 | 0 | 1097 | 0 | 0 | 0 |
| Serbia | 0 | 0 | 0 | 0 | 0 | 0 | 0 | 0 | 800 | 0 | 0 | 0 |
| Slovakia | 0 | 0 | 0 | 0 | 0 | 0 | 0 | 130 | 0 | 0 | 0 | 0 |
| Slovenia | 133 | 220 | 302 | 0 | 554 | 3965 | 6202 | 3697 | 1394 | 42 | 0 | 949 |
| Spain | 11868 | 220 | 439 | 5089 | 16456 | 22862 | 18060 | 24918 | 22735 | 6062 | 843 | 491 |
| Sweden | 0 | 36 | 712 | 12802 | 19627 | 5166 | 267 | 1175 | 41632 | 92200 | 15136 | 357 |
| Switzerland | 0 | 0 | 57 | 29 | 0 | 0 | 0 | 0 | 0 | 0 | 1636 | 0 |
