# Supplementary material for: Availability and representativeness of freshwater macroinvertebrate data to enable Europe-wide assessment of biodiversity trends
Source: PLoS One. 2026 Aug 13;21(8):e0354723. doi: 10.1371/journal.pone.0354723 (PMC13472404; doi:10.1371/journal.pone.0354723)
Supplement: Supplementary file 8 [file pone.0354723.s009.docx]

**S2 File. Answers to the questionnaire Part 2 and Part 3.**

**PART 2: Biodiversity data**

Information on the methodology

1. Does the monitoring data consist of raw Water Framework Directive (WFD) data?
   a) **Yes**. Total: 31
   b) **Partly**. Total: 10
   c) **No**. Total: 5
2. Was the fieldwork conducted following a WFD protocol?
a) **Yes, the protocol used is according to the WFD, but was adjusted to the existing monitoring scheme in my country.** Total: 34
b) **No, a different kind of protocol was used.** Total: 5
c) **Other.** Total: 6

- "Both"
- "Field work did not strictly follow WFD protocol, but rather similar anyhow"
- "It was only for wadable rivers"
- “WFD doesn't have protocols. International standards are used.”
- "Yes"
- “Yes, the AQEM protocol”

3. Was sampling performed differently depending on the size of the stream?
a) **Yes.** Total: 13
b) **No.** Total: 30
4. The sampling was performed differently depending on the size of the stream. Please elaborate (e.g. a small stream was considered..):
   a) **Small Streams.** Total: 2
      - “Sampling typically involves a shorter duration of kicknet sampling,

around 3 minutes.”

- “Sampling is conducted in spring.”
- **Large Streams.** Total: 5
  - “Sampling duration for kicknet may be longer, approximately 5

minutes.”

- “Some larger streams utilize artificial substrates or grab samplers instead of kicknets, especially where kicknet sampling is not feasible.”
- “Sampling is usually done in summer.”
- “In some cases (e.g., federal waterways with shipping concerns), baskets with typical stream substrate are used; about 8 baskets are distributed, covering a total area of 1 m².”
- “The sampling method may vary but often includes methods like PTI (for gravel in low mountain range streams) or PTI and AeTV(+) (for sand in lowland and tidal-influenced streams).”
- **General Methodology Across Streams.** Total: 3
- “The length of the sampled section varies based on the stream size, ranging from 25 to 250 meters.”
- “Sampling is generally conducted using the same methods regardless of stream size, but the specifics vary as described above.”
- “Typically, 1 sampling site is selected per water body, with adjustments in methodology according to the type of water body (e.g., transitional water vs. river vs. stream).”
- **Unwadable Rivers (e.g., Elbe).** Total: 1
- “The sampling methodology used is tailored for unwadable rivers, which may involve specific methods detailed for such water bodies.”

5. Was the fieldwork done using a hand net or a core sampler or both depending on the field situation?
   a) **A hand net.** Total: 38
   b) **A core sampler.** Total: 1
   c) **Both were used.** Total: 4
   d) **Other.** Total: 1

- “At pool sections samples were taken using Ekman grab sampler, but at riffle section using Surber Sampler”

6. Does one sample consists of an average of multiple subsamples?
   a) **Yes, according to the multihabitat sampling (MHS) scheme.** Total: 25
   b) **Yes.** Total: 12
   c) **No.** Total: 6
   d) **Other.** Total: 6

-	“Multihabitat sampling was done but restricted to wadable part of river.”

-	“One sample consists of 5 replications (a´0.12 m²) and of qualitative sample.”

-	“Habitats were sampled in proportion to their occurrence at the site.”

-	“We target riffle sections of the river (dominated by gravel and stones). Therein, we sample habitats in relative proportion of their occurrence.”

-	“It is a semi-quantitative method (3 min kick sampling).”

-	“For macro-invertebrates: 1 sample, for macrophytes: 3 river stretches.”

7. What is the number of subsamples per sample? If this number varies, please describe why.

-	**5 subsamples.** Total: 3

-	**12 subsamples.** Total: 2

-	**3 subsamples.** Total: 3

-	**6 subsamples.** Total: 1

-	**4 subsamples.** Total: 1

8. Would it be possible to adjust your field work and lab work approach for one specific EU wide strict protocol (i.e. determination at one specific taxon level, using one specific methodology like core sampler) in order to make data more comparable on a regional scale? Please elaborate.

-	Total: 18, **indicate that it is not feasible to adjust field work and lab work approaches to a single EU-wide strict protocol, due to various constraints.**

-	Total: 15, **believe that it is possible to adjust methodologies, indicating willingness or potential for alignment.**

-	Total: 13, **provided conditional or uncertain responses, suggesting that adjustments might be possible under certain conditions or with reservations.**

9. Can I make the macroinvertebrate data that you shared with me publicly available in .xlsx form after publishing? I will send the final sheet form for approvement.
   a) **Yes.** Total: 34
   b) **Yes, but only after a certain time - Please specify the time frame after which the data can be made available.** Total: 3
   c) **No - Please elaborate why the data cannot become available.** Total: 9

- "The data may not be published, because it contains taxa lists, which we give out only on request and have not published ourselves.”
- “Specific form-related restrictions.”
- “Permission required from state enterprise.”
- “Already published data.”
- "The raw data have been generated by many different projects and actors in the sampling, lab processing, and posterior curation. Thus, data-use should follow a strict protocol for acknowledging the funds and work of others. This is not achieved if the data are made public without any protocol. This is especially relevant for long-term data records."
- "Needs to be requested from the ministry." Total: 3
- "We spent enormous time and effort to gather the dataset and we don't want to lose control of its future use."

**PART 3: Data sharing**

information on why and how biodiversity data are being shared

1. Are you familiar with the FAIR (Findable, Accessible, Interoperable, and Reusable) data principles?
   a) **Yes.** Total: 31
   b) **No.** Total: 12
   c) **Other.** Total: 2

-	“only in restricted scope”

-	“I can guess what this is about”

2. Is data sharing being facilitated within your work environment through for example data management training, funds and/or time?
a) **Yes.** Total: 23
b) **No, but we are expected to do it in our own time.** Total: 7
c) **No, nothing is expected of me to share any data.** Total: 11
d) **Other.** Total 5:

-	“After ministerial authorization of the data, they are available on the Internet in accordance with the Environmental Information Act (assessment results) or can be requested from us if required (taxa lists).”

-	“No, we are expected to do provide data for all enquiries since the data is provided by public funding.”

-	“Our databases are publicly available”

-	“Data-sharing is increasing in many areas, however, this is still in its infancy until clear rules are established for the use and reuse of datasets whose generation have asymmetric costs. I think fair scientific collaboration will strengthen data sharing, and there is a lot to do in this regard. “

-	“I don't know“

3. How is data sharing being facilitated?

-	**Through public access, trainings, videos.** Total: 11

-	**Internal Databases and Institutional Repositories.** Total: 6

-	**Scientific Papers and Data Papers.** Total: 3

-	**Training and courses:** Total: 4

4. Are you encouraged to archive the data using a standardised template (e.g. a defined spreadsheet like the Darwin Core template from gbif)?
   a) **Yes, to help create accessible data for the public.** Total: 7
   b) **Yes, but only for the Water Framework Directive (WFD).** Total: 4
   c) **No, I use my own approach.** Total: 5
   d) **Other.** Total: 0
5. How is your data available (if multiple, please use 'other' and describe the two or more options):
   a) **By contacting me directly.** Total: 17
   b) **Via a national database**. Total: 13
   c) **Via an international database**. Total: 2
   d) **Via a data repository.** Total: 11
   e) **Other.** Total: 5

- “The provided data was originally compiled for an article”
- “personal contact or data repository”
- “As supplementary information of previous published papers”

6. What kind of data are available when present in a database or data repository?
a) **All raw data, including data of less quality (e.g. data that is not checked).**
   a. Total: 4
b) **Only raw data that is checked and of sufficient quality**. Total: 34
c) **Adjusted data, for example only ecological status data or occurrence data**. Total: 4
d) **I don’t have data available in a database.** Total: 3
7. When you are hesitant to share data, how would you become more encouraged? Multiple answers are possible:
   a) **When I trust the data is used with care.** Total: 22
   b) **When the person asking contains a high profile (e.g. a professor).** Total: 4
   c) **When I will be credited as co-author of a scientific article.** Total: 19
   d) **When I will be acknowledged in the acknowledgements of a scientific article.** Total: 10
   e) **I do not have an opinion about this because our work policy is to always share data.** Total: 10
   f) **Other**. Total: 4

- “When the data is used to promote better and more informed decision making for biodiversity and environmental protection.”
- “When it is a shared publication where datasets of similar costs are shared. “
- “It depends on the type of data, their costs to be generated, and the possibilities of publishing those datasets ourselves.”
- “We have an open data policy.”

**PART 3: Data sharing** *(complaints and improvements)*

With biodiversity being a very hot topic, sharing of monitoring data is encouraged. For example, funds are only given when data is shared at the end of the project, or journals will only publish manuscripts when data are made available. However, there seems to be a gap between policy, research and practice.

1. Do you have any complaints on how the system on sharing data is currently being executed:
   a) **No**. Total: 18
   b) **Yes**. Total: 18

- ”Yes, but mainly because of lack of a proper database and tools for the public to find the biological data and download it. This goes also for the majority of other EU countries.”
- “In Germany there are discussions about developing a database for the biological data of all federal states. The financial and personnel effort for this is high and should largely be provided by the federal states. In particular, federal states with their own databases and data portals, such as Saxony-Anhalt, are not currently considering this proposal”.
- “there should be a stringent european format for every data in context to WFD”
- “Main complaint is that much data is not accessible”
- “I think that your statement above is true only in case the data were made within the project. If you work with the data of third parties you can not be expected to share all data. Despite this fact using this data is very valuable and contributive.”
- “We have to be careful that collection of raw data (biodiversity data) is rewarded, as this data is essential to be continued to be collected. Sharing is very important, but only if original data collectors and handlers get appropriate rewards that justifies and acknowledges their work.”
- “It's insufficient. I support a European (EEA) database with WFD raw data (BQE and metadata such as water chemistry, e.g. the WISER database (which is however a bit insufficient).”
- “I do not think it is completely fair at the moment. Not all teams have the same access to research resources and, thus, data sharing becomes a winner for some and a negative issue for others. Generating ecological data is very costly, it should be rewarded, otherwise there will not be a huge increase on this practice. Scientific collaborations (in the form of papers) is a generally accepted pay-back for data generation, but other ways could be put in place.”
- “Although sharing data is vital for many reasons, I understand that there is a lot of effort (time and money) for producing the data and that the data owners want some kind of credit and control over the data.”
- “Good data sharing requires staff that are trained and have the time to commit to developing databases etc. We don't have that in our Institute, so it's mainly on individuals to get data shared, usually via suboptimal methods (e.g., excel sheets)”
- “there is no requirement to publish (I prefer the term "publish" over "sharing" as it normally includes recognition) raw data of WFD monitoring”
- “My funding doesn't ask for open access data, and I don't see the reason to share it with others without co-authorship when I spent more than 10 years (sometimes without any funds, doing it voluntarily) to collect them in the best available quality.”
- “It is too complicated to download data from the Norwegian national database for WFD data. Also, it is not easy to use the database for persons that do not speak Norwegian.”
- “Usually there is not enough metadata describing the methods used for data collection and what it represents, neither on how outliers or rare species have been treated”
- “Yes, costs coverage is mostly lacking, and inside location knowledge is not or insufficiently used”
- “Yes, there are a lot of "work-arounds" to actually being able to NOT share data.”
- “In the UK, while the data sharing by my employer, the Environment Agency for England, works well, it's harder to get data from our counterparts in Wales and Scotland. In my opinion, data exchange between these three areas is weak, and this area needs to be developed by creating platforms that would promote it.”
- “Yes, currently many project proposals include compliance with FAIR principles as one of the mandatory criteria.”

4.	Would you have any suggestions on how to change the data sharing system through e.g. time, funds, education, co-authorship, etc.? What do you think is needed for you as a data holder?

-	**Nationwide and EU-wide Data Management.** Total: 6

- “Nationwide collected data should follow clear storage rules and format guidelines (F.A.I.R. principles in general) and should be readily accessible in a public database. This would save a lot of time for data generators.”
- “Across the EU, all WFD data holders (e.g., agencies, ministries) should make biological data easily accessible.”
- “Central national data repositories are needed, along with funding for collecting faunistic field data.”
- “All WFD data in Europe should have a common platform for sharing data. Funds to share WFD data on a European platform could be available from the EU.”
- “There should be a stringent European format for every data in context to WFD.”
- “There should be a limited number of repositories that hold all data and provide a basic questionnaire with items regarding data origin, methods for their generation, etc. Standardization of the data archiving and sharing process is essential.”

-	**Improving Access and Usability**. Total: 2

- “Acknowledgement should be improved - like repository with Impact factor and citation.”
- “Easy-to-use and standardized approaches for uploading data.”

-	**Funding and Resources.** Total: 3

- “Funds that buy datasets to liberate them (though risky because they might become "privatized").”
- “Funding dedicated staff with data management skills.”
- “Funding is necessary to prepare high-quality data for sharing.”

-	**Recognition and Incentives.** Total: 5

- “Co-authorship is the most often used method for data sharing because of the huge competition for getting a scientific position and professional development.”
- “Recognition for sharing data should be equivalent to publishing papers and obtaining grants.”
- “Sharing of data should be a valuable activity in terms of career acknowledgment, given the resources and time spent on that.”
- “Particularly for long-term data, offering co-authorship should be key.”
- “Such participation with data in the writing of publications with co-authorship motivates to improve the database, continue long-term research, and provides support in project applications for obtaining funding for the continuation of the research project (long-term study).”

-	**Quality and Control**. Total: 3

- “When collating data, some central organ with gate-keeper capacity is essential to guarantee data quality.”
- “We should be careful because we are using data that we are not familiar with, and there are many particularities (e.g., methods, site) that we might be unaware of and that affect the interpretation of the results. The data need to be used with caution and understanding its limitations.”
- “Usually, there is not enough metadata describing the methods used for data collection and what it represents, neither on how outliers or rare species have been treated.”
