# Supplementary material for: Availability and representativeness of freshwater macroinvertebrate data to enable Europe-wide assessment of biodiversity trends
Source: PLoS One. 2026 Aug 13;21(8):e0354723. doi: 10.1371/journal.pone.0354723 (PMC13472404; doi:10.1371/journal.pone.0354723)
Supplement: Peer review history

# Peer review: Decision Letter 0

**DOI:** 10.1371/journal.pone.0354723.r001

**Authors:** Mattias Gaglio

11 Dec 2025

Dear Dr. Pit,

Thank you for submitting your manuscript to PLOS ONE. After careful consideration, we feel that it has merit but does not fully meet PLOS ONE’s publication criteria as it currently stands. Therefore, we invite you to submit a revised version of the manuscript that addresses the points raised during the review process.

Please submit your revised manuscript by Jan 25 2026 11:59PM. If you will need more time than this to complete your revisions, please reply to this message or contact the journal office at plosone@plos.org. When you're ready to submit your revision, log on to https://www.editorialmanager.com/pone/ and select the 'Submissions Needing Revision' folder to locate your manuscript file.

- A rebuttal letter that responds to each point raised by the academic editor and reviewer(s). You should upload this letter as a separate file labeled 'Response to Reviewers'.
- A marked-up copy of your manuscript that highlights changes made to the original version. You should upload this as a separate file labeled 'Revised Manuscript with Track Changes'.
- An unmarked version of your revised paper without tracked changes. You should upload this as a separate file labeled 'Manuscript'.

If applicable, we recommend that you deposit your laboratory protocols in protocols.io to enhance the reproducibility of your results. Protocols.io assigns your protocol its own identifier (DOI) so that it can be cited independently in the future. For instructions see: https://journals.plos.org/plosone/s/submission-guidelines#loc-laboratory-protocols. Additionally, PLOS ONE offers an option for publishing peer-reviewed Lab Protocol articles, which describe protocols hosted on protocols.io. Read more information on sharing protocols at https://plos.org/protocols?utm_medium=editorial-email&utm_source=authorletters&utm_campaign=protocols.

We look forward to receiving your revised manuscript.

Kind regards,

Mattias Gaglio, PhD

Academic Editor

PLOS One

Journal Requirements:

When submitting your revision, we need you to address these additional requirements.

1. Please ensure that your manuscript meets PLOS ONE's style requirements, including those for file naming. The PLOS ONE style templates can be found at

https://journals.plos.org/plosone/s/file?id=wjVg/PLOSOne_formatting_sample_main_body.pdf and https://journals.plos.org/plosone/s/file?id=ba62/PLOSOne_formatting_sample_title_authors_affiliations.pdf

2. Please expand the acronym “NWO” (as indicated in your financial disclosure) so that it states the name of your funders in full.

This information should be included in your cover letter; we will change the online submission form on your behalf.

3. Thank you for stating the following in the Acknowledgments Section of your manuscript:

[This research was funded by the Dutch Research Council (NWO) through the Rubicon programme (project number 019.211EN.023, “Synthetic chemicals as a cause of biodiversity loss”). The authors would like to thank the following institutions for providing biological data:

o            NLWKN – Lower Saxon State Department for Waterway, Coastal and Nature Conservation, for data collected within the context of the WFD (Water Framework Directive) monitoring.

o            LUBW – Landesanstalt für Umwelt Baden-Württemberg.

o            Slovenian Environment Agency.

o            Austrian Federal Ministry for Agriculture, Forestry, Regions and Water Management (BML, Department I/2 National and International Water Management) and the Offices of the Provincial Governments for data from the Ordinance on the Monitoring of the Quality of Water Bodies (Federal Law Gazette II No. 479/2006).

o            LHW – Landesbetrieb für Hochwasserschutz und Wasserwirtschaft Sachsen-Anhalt (State Office for Flood Protection and Water Management Saxony-Anhalt)

o            LfU Bayern – Bayerisches Landesamt für Umwelt (Bavarian Environment Agency).

o            FGG Elbe – Flussgebietsgemeinschaft Elbe (Elbe River Basin Community) (www.elbe-datenportal.de).

o            ICPDR – International Commission for the Protection of the Danube River.

o            JDS3 – Joint Danube Survey 3.

o            ICPR – International Commission for the Protection of the Rhine.

o            Ministry of Economy and Sustainable Development, Croatia.

o            Landesamt für Natur, Umwelt und Verbraucherschutz (LANUV).

o            Basque Water Agency, Spain

o            Gipuzkoa Provincial Government, Spain

o            Povodí Labe, state enterprise, Czech Republic

o            Spanish Ministry for the Ecological Transition and the Demographic Challenge

o            Environment Agency (England)

Since 16 April 2025, Miguel CANEDO ARGUELLES has been seconded to the ERC Executive agency. The views expressed in this paper are purely those of the author. They do not necessarily reflect the views or official positions of the European Commission, the ERC Executive Agency or the ERC Scientific Council.]

We note that you have provided funding information that is currently declared in your Funding Statement. However, funding information should not appear in the Acknowledgments section or other areas of your manuscript. We will only publish funding information present in the Funding Statement section of the online submission form.

Please remove any funding-related text from the manuscript and let us know how you would like to update your Funding Statement. Currently, your Funding Statement reads as follows:

[I.R. Pit

Synthetic chemicals as

a cause of biodiversity loss

(file no. 019.211EN.023)

-The grant is subject both to the Terms and Conditions of Rubicon Research Grants, round Rubicon 2021-1 S, to the NWO Regulation on Granting and The Dutch General Administrative Law Act.

-http://www.nwo.nl/en

-The sponsors did not play any role in the study design, data collection and analysis, decision to publish or preparation of the manuscript.]

Please include your amended statements within your cover letter; we will change the online submission form on your behalf.

4. We note that you have indicated that there are restrictions to data sharing for this study. PLOS only allows data to be available upon request if there are legal or ethical restrictions on sharing data publicly. For more information on unacceptable data access restrictions, please see http://journals.plos.org/plosone/s/data-availability#loc-unacceptable-data-access-restrictions.

Before we proceed with your manuscript, please address the following prompts:

a) If there are ethical or legal restrictions on sharing a de-identified data set, please explain them in detail (e.g., data contain potentially identifying or sensitive patient information, data are owned by a third-party organization, etc.) and who has imposed them (e.g., a Research Ethics Committee or Institutional Review Board, etc.). Please also provide contact information for a data access committee, ethics committee, or other institutional body to which data requests may be sent.

b) If there are no restrictions, please upload the minimal anonymized data set necessary to replicate your study findings to a stable, public repository and provide us with the relevant URLs, DOIs, or accession numbers. For a list of recommended repositories, please see

https://journals.plos.org/plosone/s/recommended-repositories. You also have the option of uploading the data as Supporting Information files, but we would recommend depositing data directly to a data repository if possible.

We will update your Data Availability statement on your behalf to reflect the information you provide.

5. We note that Figures 3, 4, and S4 in your submission contain map images which may be copyrighted. All PLOS content is published under the Creative Commons Attribution License (CC BY 4.0), which means that the manuscript, images, and Supporting Information files will be freely available online, and any third party is permitted to access, download, copy, distribute, and use these materials in any way, even commercially, with proper attribution. For these reasons, we cannot publish previously copyrighted maps or satellite images created using proprietary data, such as Google software (Google Maps, Street View, and Earth). For more information, see our copyright guidelines: http://journals.plos.org/plosone/s/licenses-and-copyright.

We require you to either (1) present written permission from the copyright holder to publish these figures specifically under the CC BY 4.0 license, or (2) remove the figures from your submission:

1. You may seek permission from the original copyright holder of Figures 3, 4, and S4 to publish the content specifically under the CC BY 4.0 license.

We recommend that you contact the original copyright holder with the Content Permission Form (http://journals.plos.org/plosone/s/file?id=7c09/content-permission-form.pdf) and the following text:

“I request permission for the open-access journal PLOS ONE to publish XXX under the Creative Commons Attribution License (CCAL) CC BY 4.0 (http://creativecommons.org/licenses/by/4.0/). Please be aware that this license allows unrestricted use and distribution, even commercially, by third parties. Please reply and provide explicit written permission to publish XXX under a CC BY license and complete the attached form.”

Please upload the completed Content Permission Form or other proof of granted permissions as an "Other" file with your submission.

In the figure caption of the copyrighted figure, please include the following text: “Reprinted from [ref] under a CC BY license, with permission from [name of publisher], original copyright [original copyright year].”

2. If you are unable to obtain permission from the original copyright holder to publish these figures under the CC BY 4.0 license or if the copyright holder’s requirements are incompatible with the CC BY 4.0 license, please either i) remove the figure or ii) supply a replacement figure that complies with the CC BY 4.0 license. Please check copyright information on all replacement figures and update the figure caption with source information. If applicable, please specify in the figure caption text when a figure is similar but not identical to the original image and is therefore for illustrative purposes only.

The following resources for replacing copyrighted map figures may be helpful:

USGS National Map Viewer (public domain): http://viewer.nationalmap.gov/viewer/

The Gateway to Astronaut Photography of Earth (public domain): http://eol.jsc.nasa.gov/sseop/clickmap/

Maps at the CIA (public domain): https://www.cia.gov/library/publications/the-world-factbook/index.html and https://www.cia.gov/library/publications/cia-maps-publications/index.html

NASA Earth Observatory (public domain): http://earthobservatory.nasa.gov/Landsat: http://landsat.visibleearth.nasa.gov/

USGS EROS (Earth Resources Observatory and Science (EROS) Center) (public domain): http://eros.usgs.gov/#

Natural Earth (public domain): http://www.naturalearthdata.com/

If the reviewer comments include a recommendation to cite specific previously published works, please review and evaluate these publications to determine whether they are relevant and should be cited. There is no requirement to cite these works unless the editor has indicated otherwise.

Please review your reference list to ensure that it is complete and correct. If you have cited papers that have been retracted, please include the rationale for doing so in the manuscript text, or remove these references and replace them with relevant current references. Any changes to the reference list should be mentioned in the rebuttal letter that accompanies your revised manuscript. If you need to cite a retracted article, indicate the article’s retracted status in the References list and also include a citation and full reference for the retraction notice.

[Note: HTML markup is below. Please do not edit.]

Reviewers' comments:

Reviewer's Responses to Questions

**Comments to the Author**

1. Is the manuscript technically sound, and do the data support the conclusions?

Reviewer #1: Yes

Reviewer #2: No

**********

2. Has the statistical analysis been performed appropriately and rigorously?

Reviewer #1: Yes

Reviewer #2: Yes

**********

3. Have the authors made all data underlying the findings in their manuscript fully available?

The PLOS Data policy

Reviewer #1: Yes

Reviewer #2: No

**********

4. Is the manuscript presented in an intelligible fashion and written in standard English?

Reviewer #1: Yes

Reviewer #2: Yes

**********

Reviewer #1: Pit and colleagues show the heterogeneity (and homogeneity) of available European macroinvertebrate monitoring data and present the sentiments of freshwater data curators and providers toward potentially standardizing European sampling. Given the increasing importance of big data, this is substantial achievement, especially since they present FAIR data for 28 European countries! This will certainly be useful for future meta-analyses and syntheses. However, being a curator of data from Lithuania and Poland, I am surprised that these data were not included, especially since several colleagues on the manuscript are aware that such data from these countries exist. Specifically, Lithuania has macroinvertebrate data for 462 river sites collected between 2010 – 2016 (25.76% have a single year of sampling, 45.24% have 2 years sampling, 17.75% have three years, with 52 sites having five years or more of sampling), and for 253 lake sites collected between 2013 – 2016 (90.51% only have a single year of data). In Poland, macroinvertebrate data are available from 710 river sites (87.18% only have a single year of sampling, 11.55% have two years of sampling) and 26 lake sites (100% have data for a single year) for the years 2010 – 2016. Neither the Lithuanian nor the Polish data were included in Welti et al. (2024) – Scientific data, but subsets of the Lithuanian data have been included in other manuscripts, namely Sinclair et al. (2024) – Nature Ecology and Evolution and Baker et al. (2025) – Hydrobiologia. I do, however, understand and acknowledge the potential conflict of interest here. While it would allow for greater spatial representatitiveness of the current manuscipt, I do not explicitly advocate for including these data, I merely wanted to bring it to the Authors’ attention. With that said, the data inclusion criteria are unclearly stipulated within the presented manuscript and I recommend the authors spend a few sentences describing them in more detail. For example: Did the data have to be sampled in each year between 2010 and 2015? Were there a minimum number of sites required per country? Note: This comment alos pertains to a comment further down highlighting discrepensicies in data availability between countries.

The authors open the floor to an interesting topic, namely the potential of a “strict EU-wide protocol” for invertebrate sampling. I must admit that I too have my reservations about its feasibility due to, amongst other things, the impact it might have on the homogeneity of long-term monitoring programs at the national level (there were already observed shifts in German monitoring around 2004) but it is nonetheless an important conversation to have. Moreover, the authors advocate for collecting species level data where possible but offer no suggestions on how to improve traditional (i.e., non-DNA-based) identification, as it remains time consuming and difficult, especially for mega-diverse groups like Diptera. DNA-based identification methods can be used as a complement to traditional identification, but I also believe that the development of an EU-defined standardized operational list is just as pertinent and should be added to the discussion. The Water Framework Directive seeks harmonized biomonitoring approaches across Europe but lacks explicit taxonomic requirements for biological quality elements. This gap allows member states to independently define macroinvertebrate operational taxonomic units, creating taxonomic inconsistencies across national and regional scales. While the authors acknowledge that freshwaterecology.info is a possible tool, one I use regularly myself, it still contains incongruencies and is optimized for western and central European fauna.

Other comments (Note: The lack of line and page numbers makes pinpointing exact locations within the manuscript difficult):

Material and Methods, section 2.1: It is unclear why the WISE database was used during the initial phase of data aggregation, as it only provides the final EQR value. Please elaborate as to why it was used if the goal was to aggregate raw data.

Material and Methods, section 3.2: You state that no data are available from Slovakia, but later you present data from Slovakia. Please correct accordingly.

Results, section 3.3: These data are highly dependent on the number of institutions in any given country that have access to it and that conduct macroinvertebrate surveys. The more institutions, the greater the likelihood of more sampling points. It is therefore unclear how many institutions in each country were selected, and whether comparability can truly be achieved. For instance, very few data points are available for Latvia, with these points not accurately reflecting the monitoring network across the entire country. The data exists. Therefore, statements like “For many other countries (e.g., Portugal, Switzerland, and Slovakia), only a limited amount of stream orders is represented and smaller streams (orders 1 and 2) tend to be neglected” are not well supported since the data exists, but the authors just do not have it (e.g., see recent paper by Šamulková et al. 2025 – Metabarcoding & Metagenomics).

Material and Methods, section 2.3: Another avenue for exploring the data may be to examine the time of year in which countries collect their samples (e.g., sampling season). This would provide an additional layer for the discussion around Strahler order and land use, as different countries conduct their sampling at different times of year. I assume that there will also be discrepancies here that could limit comparisons between countries.

All things considered, I do recommend this manuscript for publication in PLOS ONE after the minor points are addressed. Further, to mitigate any potential conflicts of interest, I waive my anonymity.

Sincerely,

Nathan Jay Baker

Reviewer #2: The manuscript PONE-D-25-54149 titled “Availability and representativeness of freshwater macroinvertebrate data to enable Europe-wide assessment of biodiversity trends” studied macroinvertebrate diversity trends in freshwater ecosystem over a period of 15 years. The authors stressed the importance of harmonizing data standardization in relation to taxonomic resolution and sampling efforts that will facilitate biodiversity conservation efforts.

The manuscript addresses a true challenge in research - data harmonization and has worked to provide suggestions regarding the same. It is a herculean task and the authors have done a commendable job indeed.

However, as pointed by the authors in the manuscript, not everyone ma be willing to share data, and even ion they do so, may not provide permission to make it accessible to the public. As mentioned, close to almost half the data used in this work was acquired by direct contact with the contributors. The question of harmonization comes into the circle only when the data is available.

Moreover, there are often barriers - institutional or otherwise including, but not limited to funding agencies - regarding data distribution. These are some real world challenges that require answering.

On the varying taxonomic level, the detail to which data is often collected may not be properly reflected - for example, only families may be mentioned and not genus or species. The main problem in harmonizing these type of raw data lies in the inconsistency of resolution.

Sampling effort and protocol, seasonality are some of the facets to consider while collecting the data. It affects the data density (as reported in table 2) and can create inaccuracies regarding the reliability.

These are a few concerns that came to mind when reading the manuscript, and may be beneficial to add/justify them.

**********

what does this mean?). If published, this will include your full peer review and any attached files.

If you choose “no”, your identity will remain anonymous but your review may still be made public.

**Do you want your identity to be public for this peer review?**  For information about this choice, including consent withdrawal, please see our Privacy Policy

Reviewer #1: **Yes:**  Nathan Jay Baker

Reviewer #2: No

**********

[NOTE: If reviewer comments were submitted as an attachment file, they will be attached to this email and accessible via the submission site. Please log into your account, locate the manuscript record, and check for the action link "View Attachments". If this link does not appear, there are no attachment files.]

To ensure your figures meet our technical requirements, please review our figure guidelines: https://journals.plos.org/plosone/s/figures

You may also use PLOS’s free figure tool, NAAS, to help you prepare publication quality figures: https://journals.plos.org/plosone/s/figures#loc-tools-for-figure-preparation.

NAAS will assess whether your figures meet our technical requirements by comparing each figure against our figure specifications.

# Author comment: Author response to Decision Letter 1

**DOI:** 10.1371/journal.pone.0354723.r002

25 Jun 2026

Response to Reviewers

Manuscript PONE-D-25-54149

"Availability and representativeness of freshwater macroinvertebrate data to enable Europe-wide assessment of biodiversity trends"

We thank the editor and reviewers for their constructive and insightful feedback. Below we address each comment point by point. Any changes to the manuscript are described which section and visible in the manuscript via track-changes.

Journal Requirements

When submitting your revision, we need you to address these additional requirements.

1. Please ensure that your manuscript meets PLOS ONE's style requirements, including those for file naming.

We have reviewed the manuscript against the PLOS ONE style templates and made the following adjustments: (1) "Summary" has been renamed to "Abstract", (2) section numbering has been removed, (3) keywords have been removed from the manuscript, (4) the reference style has been updated to numbered format, (5) figure captions have been formatted according to PLOS ONE guidelines, and (6) all figures have been saved as TIFF files with the required naming convention.

2. Please expand the acronym “NWO” (as indicated in your financial disclosure) so that it states the name of your funders in full.

The full name of the funder is: Nederlandse Organisatie voor Wetenschappelijk Onderzoek (Dutch Research Council, NWO). The amended Funding Statement has been included in the cover letter.

3. Thank you for stating the following in the Acknowledgments Section of your manuscript:

[This research was funded by the Dutch Research Council (NWO) through the Rubicon programme (project number 019.211EN.023, “Synthetic chemicals as a cause of biodiversity loss”). The authors would like to thank the following institutions for providing biological data:

o NLWKN – Lower Saxon State Department for Waterway, Coastal and Nature Conservation, for data collected within the context of the WFD (Water Framework Directive) monitoring.

o LUBW – Landesanstalt für Umwelt Baden-Württemberg.

o Slovenian Environment Agency.

o Austrian Federal Ministry for Agriculture, Forestry, Regions and Water Management (BML, Department I/2 National and International Water Management) and the Offices of the Provincial Governments for data from the Ordinance on the Monitoring of the Quality of Water Bodies (Federal Law Gazette II No. 479/2006).

o LHW – Landesbetrieb für Hochwasserschutz und Wasserwirtschaft Sachsen-Anhalt (State Office for Flood Protection and Water Management Saxony-Anhalt)

o LfU Bayern – Bayerisches Landesamt für Umwelt (Bavarian Environment Agency).

o FGG Elbe – Flussgebietsgemeinschaft Elbe (Elbe River Basin Community) (www.elbe-datenportal.de).

o ICPDR – International Commission for the Protection of the Danube River.

o JDS3 – Joint Danube Survey 3.

o ICPR – International Commission for the Protection of the Rhine.

o Ministry of Economy and Sustainable Development, Croatia.

o Landesamt für Natur, Umwelt und Verbraucherschutz (LANUV).

o Basque Water Agency, Spain

o Gipuzkoa Provincial Government, Spain

o Povodí Labe, state enterprise, Czech Republic

o Spanish Ministry for the Ecological Transition and the Demographic Challenge

o Environment Agency (England)

Since 16 April 2025, Miguel CANEDO ARGUELLES has been seconded to the ERC Executive agency. The views expressed in this paper are purely those of the author. They do not necessarily reflect the views or official positions of the European Commission, the ERC Executive Agency or the ERC Scientific Council.]

We note that you have provided funding information that is currently declared in your Funding Statement. However, funding information should not appear in the Acknowledgments section or other areas of your manuscript. We will only publish funding information present in the Funding Statement section of the online submission form.

Please remove any funding-related text from the manuscript and let us know how you would like to update your Funding Statement. Currently, your Funding Statement reads as follows:

[I.R. Pit

Synthetic chemicals as

a cause of biodiversity loss

(file no. 019.211EN.023)

-The grant is subject both to the Terms and Conditions of Rubicon Research Grants, round Rubicon 2021-1 S, to the NWO Regulation on Granting and The Dutch General Administrative Law Act.

-http://www.nwo.nl/en

-The sponsors did not play any role in the study design, data collection and analysis, decision to publish or preparation of the manuscript.]

Please include your amended statements within your cover letter; we will change the online submission form on your behalf.

We have removed the funding-related text ("This research was funded by the Dutch Research Council (NWO) through the Rubicon programme...") from the Acknowledgments section. The remaining acknowledgments, including the list of data-providing institutions and the ERC disclaimer, have been retained. The updated Funding Statement has been included in the cover letter for the editor to update the online submission form.

4. We note that you have indicated that there are restrictions to data sharing for this study. PLOS only allows data to be available upon request if there are legal or ethical restrictions on sharing data publicly. For more information on unacceptable data access restrictions, please see http://journals.plos.org/plosone/s/data-availability#loc-unacceptable-data-access-restrictions.

Before we proceed with your manuscript, please address the following prompts:

a) If there are ethical or legal restrictions on sharing a de-identified data set, please explain them in detail (e.g., data contain potentially identifying or sensitive patient information, data are owned by a third-party organization, etc.) and who has imposed them (e.g., a Research Ethics Committee or Institutional Review Board, etc.). Please also provide contact information for a data access committee, ethics committee, or other institutional body to which data requests may be sent.

b) If there are no restrictions, please upload the minimal anonymized data set necessary to replicate your study findings to a stable, public repository and provide us with the relevant URLs, DOIs, or accession numbers. For a list of recommended repositories, please see

https://journals.plos.org/plosone/s/recommended-repositories. You also have the option of uploading the data as Supporting Information files, but we would recommend depositing data directly to a data repository if possible.

We will update your Data Availability statement on your behalf to reflect the information you provide.

89% of the compiled dataset has been made publicly available via Zenodo (https://doi.org/10.5281/zenodo.15268354) under a CC BY 4.0 license. The remaining 11% cannot be made publicly available due to restrictions imposed by the data owners. These restrictions include: (1) data ownership by third-party organizations (e.g., state agencies) that do not permit redistribution, (2) data contributors who require controlled access to ensure proper acknowledgment of the original data collection efforts, and (3) data contributors from whom explicit consent for public sharing could not be obtained. Researchers wishing to access the restricted data may contact Iris Pit (iris.pit@deltares.nl), who can facilitate requests to the respective data owners.

5. We note that Figures 3, 4, and S4 in your submission contain map images which may be copyrighted. All PLOS content is published under the Creative Commons Attribution License (CC BY 4.0), which means that the manuscript, images, and Supporting Information files will be freely available online, and any third party is permitted to access, download, copy, distribute, and use these materials in any way, even commercially, with proper attribution. For these reasons, we cannot publish previously copyrighted maps or satellite images created using proprietary data, such as Google software (Google Maps, Street View, and Earth). For more information, see our copyright guidelines: http://journals.plos.org/plosone/s/licenses-and-copyright.

We require you to either (1) present written permission from the copyright holder to publish these figures specifically under the CC BY 4.0 license, or (2) remove the figures from your submission:

1. You may seek permission from the original copyright holder of Figures 3, 4, and S4 to publish the content specifically under the CC BY 4.0 license.

We recommend that you contact the original copyright holder with the Content Permission Form (http://journals.plos.org/plosone/s/file?id=7c09/content-permission-form.pdf) and the following text:

“I request permission for the open-access journal PLOS ONE to publish XXX under the Creative Commons Attribution License (CCAL) CC BY 4.0 (http://creativecommons.org/licenses/by/4.0/). Please be aware that this license allows unrestricted use and distribution, even commercially, by third parties. Please reply and provide explicit written permission to publish XXX under a CC BY license and complete the attached form.”

Please upload the completed Content Permission Form or other proof of granted permissions as an "Other" file with your submission.

In the figure caption of the copyrighted figure, please include the following text: “Reprinted from [ref] under a CC BY license, with permission from [name of publisher], original copyright [original copyright year].”

2. If you are unable to obtain permission from the original copyright holder to publish these figures under the CC BY 4.0 license or if the copyright holder’s requirements are incompatible with the CC BY 4.0 license, please either i) remove the figure or ii) supply a replacement figure that complies with the CC BY 4.0 license. Please check copyright information on all replacement figures and update the figure caption with source information. If applicable, please specify in the figure caption text when a figure is similar but not identical to the original image and is therefore for illustrative purposes only.

The following resources for replacing copyrighted map figures may be helpful:

USGS National Map Viewer (public domain): http://viewer.nationalmap.gov/viewer/

The Gateway to Astronaut Photography of Earth (public domain): http://eol.jsc.nasa.gov/sseop/clickmap/

Maps at the CIA (public domain): https://www.cia.gov/library/publications/the-world-factbook/index.html and https://www.cia.gov/library/publications/cia-maps-publications/index.html

NASA Earth Observatory (public domain): http://earthobservatory.nasa.gov/Landsat: http://landsat.visibleearth.nasa.gov/

USGS EROS (Earth Resources Observatory and Science (EROS) Center) (public domain): http://eros.usgs.gov/#

Natural Earth (public domain): http://www.naturalearthdata.com/

If the reviewer comments include a recommendation to cite specific previously published works, please review and evaluate these publications to determine whether they are relevant and should be cited. There is no requirement to cite these works unless the editor has indicated otherwise.

Please review your reference list to ensure that it is complete and correct. If you have cited papers that have been retracted, please include the rationale for doing so in the manuscript text, or remove these references and replace them with relevant current references. Any changes to the reference list should be mentioned in the rebuttal letter that accompanies your revised manuscript. If you need to cite a retracted article, indicate the article’s retracted status in the References list and also include a citation and full reference for the retraction notice.

Upon review, Figure 4 does not contain a copyrighted base map and did not require replacement. The base maps in Figures 2, 3, S1 and S2 (previously S4) have been replaced with Natural Earth data (public domain, naturalearthdata.com). The data points overlaid on the maps are our own. This has been noted in the figure captions. The supporting information has been renumbered accordingly.

Regarding the reference list, the following references have been added: Baker et al. (2025), Sinclair et al. (2024), Šamulková et al. (2025), Haase et al. (2010), Elbrecht et al. (2017). We have reviewed the reference list for completeness and correctness.

Reviewer #1

Pit and colleagues show the heterogeneity (and homogeneity) of available European macroinvertebrate monitoring data and present the sentiments of freshwater data curators and providers toward potentially standardizing European sampling. Given the increasing importance of big data, this is substantial achievement, especially since they present FAIR data for 28 European countries! This will certainly be useful for future meta-analyses and syntheses. However, being a curator of data from Lithuania and Poland, I am surprised that these data were not included, especially since several colleagues on the manuscript are aware that such data from these countries exist. Specifically, Lithuania has macroinvertebrate data for 462 river sites collected between 2010 – 2016 (25.76% have a single year of sampling, 45.24% have 2 years sampling, 17.75% have three years, with 52 sites having five years or more of sampling), and for 253 lake sites collected between 2013 – 2016 (90.51% only have a single year of data). In Poland, macroinvertebrate data are available from 710 river sites (87.18% only have a single year of sampling, 11.55% have two years of sampling) and 26 lake sites (100% have data for a single year) for the years 2010 – 2016. Neither the Lithuanian nor the Polish data were included in Welti et al. (2024) – Scientific data, but subsets of the Lithuanian data have been included in other manuscripts, namely Sinclair et al. (2024) – Nature Ecology and Evolution and Baker et al. (2025) – Hydrobiologia. I do, however, understand and acknowledge the potential conflict of interest here. While it would allow for greater spatial representatitiveness of the current manuscipt, I do not explicitly advocate for including these data, I merely wanted to bring it to the Authors’ attention. With that said, the data inclusion criteria are unclearly stipulated within the presented manuscript and I recommend the authors spend a few sentences describing them in more detail. For example: Did the data have to be sampled in each year between 2010 and 2015? Were there a minimum number of sites required per country? Note: This comment alos pertains to a comment further down highlighting discrepensicies in data availability between countries.

We thank the reviewer for the positive evaluation of our work and for the transparency regarding the Lithuanian and Polish data. We address the specific points below:

1. Lithuanian data: We are aware of the existence of macroinvertebrate monitoring data from Lithuania, as described by the reviewer and included in part in Sinclair et al. (2024). We attempted to obtain these data through collaboration with other researchers, but the original data holder did not grant permission for their use in this study. We have acknowledged this in the revised manuscript (Results, ‘General dataset information’).

2. Polish data: For Poland, only fish data were available to us through our outreach efforts; macroinvertebrate data could not be obtained. We have clarified this in the Results section ‘General dataset information’.

3. Data inclusion criteria: No minimum number of sampling sites, years, or sampling frequency per country was required. All available data within the 2010–2015 timeframe were included, regardless of the amount. We have added this clarification in the Materials and Methods under ‘General approach’.

4. Discrepancies in data availability between countries: We have revised the Results, section ‘General dataset information’ to clarify that the absence of data from certain countries reflects data sharing constraints rather than non-existence of monitoring data. We also corrected the erroneous listing of Romania and Slovakia as countries without data, as both are represented in Table 2. The number of countries has been clarified to 28 countries total, comprising 24 EU Member States and four non

Attachment Submitted filename: Response to editor and reviewers.pdf

# Peer review: Decision Letter 1

**DOI:** 10.1371/journal.pone.0354723.r003

**Authors:** Mattias Gaglio

13 Jul 2026

Availability and representativeness of freshwater macroinvertebrate data to enable Europe-wide assessment of biodiversity trends

PONE-D-25-54149R1

Dear Dr. Pit,

We’re pleased to inform you that your manuscript has been judged scientifically suitable for publication and will be formally accepted for publication once it meets all outstanding technical requirements.

Within one week, you’ll receive an e-mail detailing the required amendments. When these have been addressed, you’ll receive a formal acceptance letter and your manuscript will be scheduled for publication.

An invoice will be generated when your article is formally accepted. Please note, if your institution has a publishing partnership with PLOS and your article meets the relevant criteria, all or part of your publication costs will be covered. Please make sure your user information is up-to-date by logging into Editorial Manager at Editorial Manager® and clicking the ‘Update My Information' link at the top of the page. For questions related to billing, please contact billing support.

If your institution or institutions have a press office, please notify them about your upcoming paper to help maximize its impact. If they’ll be preparing press materials, please inform our press team as soon as possible -- no later than 48 hours after receiving the formal acceptance. Your manuscript will remain under strict press embargo until 2 pm Eastern Time on the date of publication. For more information, please contact onepress@plos.org.

Kind regards,

Mattias Gaglio, PhD

Academic Editor

PLOS One

Additional Editor Comments (optional):

Reviewers' comments:

Reviewer's Responses to Questions

**Comments to the Author**

Reviewer #1: All comments have been addressed

Reviewer #2: All comments have been addressed

**********

2. Is the manuscript technically sound, and do the data support the conclusions?

Reviewer #1: Yes

Reviewer #2: Yes

**********

3. Has the statistical analysis been performed appropriately and rigorously?

Reviewer #1: Yes

Reviewer #2: Yes

**********

4. Have the authors made all data underlying the findings in their manuscript fully available?

The PLOS Data policy

Reviewer #1: No

Reviewer #2: Yes

**********

5. Is the manuscript presented in an intelligible fashion and written in standard English?

Reviewer #1: Yes

Reviewer #2: Yes

**********

Reviewer #1: Pit and colleagues have comprehensively addressed my comments and concerns. I have no further comments and recommend that this work be accepted in its current form. Thank you for giving me the opportunity to review this timely piece of work.

Reviewer #2: All the comments from my side have been properly addressed. I have no objection to the publication of this manuscript.

**********

what does this mean?). If published, this will include your full peer review and any attached files.

If you choose “no”, your identity will remain anonymous but your review may still be made public.

**Do you want your identity to be public for this peer review?**  For information about this choice, including consent withdrawal, please see our Privacy Policy

Reviewer #1: **Yes:**  Nathan Jay Baker

Reviewer #2: No

**********

# Editor report: Acceptance letter

**DOI:** 10.1371/journal.pone.0354723.r004

**Authors:** Mattias Gaglio

PONE-D-25-54149R1

PLOS One

Dear Dr. Pit,

I'm pleased to inform you that your manuscript has been deemed suitable for publication in PLOS One. Congratulations! Your manuscript is now being handed over to our production team.

At this stage, our production department will prepare your paper for publication. This includes ensuring the following:

* All references, tables, and figures are properly cited

* All relevant supporting information is included in the manuscript submission,

* There are no issues that prevent the paper from being properly typeset

You will receive further instructions from the production team, including instructions on how to review your proof when it is ready. Please keep in mind that we are working through a large volume of accepted articles, so please give us a few days to review your paper and let you know the next and final steps.

Lastly, if your institution or institutions have a press office, please let them know about your upcoming paper now to help maximize its impact. If they'll be preparing press materials, please inform our press team within the next 48 hours. Your manuscript will remain under strict press embargo until 2 pm Eastern Time on the date of publication. For more information, please contact onepress@plos.org.

You will receive an invoice from PLOS for your publication fee after your manuscript has reached the completed accept phase. If you receive an email requesting payment before acceptance or for any other service, this may be a phishing scheme. Learn how to identify phishing emails and protect your accounts at https://explore.plos.org/phishing.

If we can help with anything else, please email us at customercare@plos.org.

Thank you for submitting your work to PLOS ONE and supporting open access.

Kind regards,

PLOS ONE Editorial Office Staff

on behalf of

Dr. Mattias Gaglio

Academic Editor

PLOS One
